# Assessing the Likelihood of Gene Flow From Sugarcane (*Saccharum* Hybrids) to Wild Relatives in South Africa

**DOI:** 10.3389/fbioe.2018.00072

**Published:** 2018-06-07

**Authors:** Sandy J. Snyman, Dennis M. Komape, Hlobisile Khanyi, Johnnie van den Berg, Dirk Cilliers, Dyfed Lloyd Evans, Sandra Barnard, Stefan J. Siebert

**Affiliations:** ^1^Crop Biology Resource Centre, South African Sugarcane Research Institute, Mount Edgecombe, South Africa; ^2^Department of Biology, School of Life Sciences, University of KwaZulu-Natal, Westville, South Africa; ^3^Unit for Environmental Sciences and Management, North-West University, Potchefstroom, South Africa; ^4^BeauSci Ltd., Waterbeach, Cambridge, United Kingdom

**Keywords:** gene flow, hybridization, pollen viability, phytogeography, spatial assessment, phylogeny, *Miscanthidium*

## Abstract

Pre-commercialization studies on environmental biosafety of genetically modified (GM) crops are necessary to evaluate the potential for sexual hybridization with related plant species that occur in the release area. The aim of the study was a preliminary assessment of factors that may contribute to gene flow from sugarcane (*Saccharum* hybrids) to indigenous relatives in the sugarcane production regions of Mpumalanga and KwaZulu-Natal provinces, South Africa. In the first instance, an assessment of *Saccharum* wild relatives was conducted based on existing phylogenies and literature surveys. The prevalence, spatial overlap, proximity, distribution potential, and flowering times of wild relatives in sugarcane production regions based on the above, and on herbaria records and field surveys were conducted for *Imperata, Sorghum, Cleistachne*, and *Miscanthidium* species. Eleven species were selected for spatial analyses based on their presence within the sugarcane cultivation region: four species in the Saccharinae and seven in the Sorghinae. Secondly, fragments of the nuclear internal transcribed spacer (ITS) regions of the 5.8s ribosomal gene and two chloroplast genes, ribulose-bisphosphate carboxylase (*rbcL*), and maturase K (*matK*) were sequenced or assembled from short read data to confirm relatedness between *Saccharum* hybrids and its wild relatives. Phylogenetic analyses of the ITS cassette showed that the closest wild relative species to commercial sugarcane were *Miscanthidium capense, Miscanthidium junceum*, and *Narenga porphyrocoma. Sorghum* was found to be more distantly related to *Saccharum* than previously described. Based on the phylogeny described in our study, the only species to highlight in terms of evolutionary divergence times from *Saccharum* are those within the genus *Miscanthidium*, most especially *M. capense*, and *M. junceum* which are only 3 million years divergent from *Saccharum*. Field assessment of pollen viability of 13 commercial sugarcane cultivars using two stains, iodine potassium iodide (IKI) and triphenyl tetrazolium chloride, showed decreasing pollen viability (from 85 to 0%) from the north to the south eastern regions of the study area. Future work will include other aspects influencing gene flow such as cytological compatibility and introgression between sugarcane and *Miscanthidium* species.

## Introduction

Commercial sugarcane (*Saccharum* hybrids) was thought to have arisen from an interspecific hybridization event between *S. spontaneum* and *S. officinarum* in Java in the late 1800's (Paterson et al., 2013). Recent literature, though, suggests that the heritage is more complicated, especially when considering the nuclear phyologenetic relationships (Lloyd Evans and Joshi, 2016a). The complex ancestry, the polyploid and aneuploid nature of modern sugarcane makes conventional breeding challenging (Butterfield et al., 2001). Notwithstanding these issues, in excess of 60 “*N*” sugarcane cultivars have been released in the South African industry since 1955, but environmental constraints affect sexual hybridization because floral induction, flowering synchronicity between selected parental germplasm and pollen fertility are problematic at sub-tropical latitudes (Brett, 1950; Horsley and Zhou, 2013). Attempts to increase genetic diversity by intergeneric crossing of commercial hybrids and members of the “*Saccharum* complex” have met with either limited or no success, even under controlled conditions with human intervention, and there are no reports of such hybridization in the wild (Bonnett et al., 2008; Cheavegatti-Gianotto et al., 2011; Organisation for Economic Cooperation and Development, 2013).

Cultivar improvement using genetic modification (GM) technology is being explored and a range of traits have been introduced to sugarcane (reviews by Lakshmanan et al., 2005; Brumbley et al., 2008; Meyer and Snyman, 2013). Commercial cultivation of GM sugarcane has only been approved in Indonesia (Xue et al., 2014) and more recently, Brazil^1^, but research of this nature is underway in most sugarcane-producing countries.

In South Africa, legislation governs the use and cultivation of GM crops [namely the Genetically Modified Organisms Act (Act 15 of 1997) and the National Environmental Management Act (Act 107 of 1998)]. One aspect of GM crop cultivation that requires assessment prior to commercial release is establishing the likelihood of lateral gene flow between related plant species. Hybridization is only possible between a crop plant and a wild relative if a number of barriers to gene flow are traversed (McGeoch et al., 2009). According to den Nijs et al. (2004), successful gene transfer (barrier crossing) requires plant populations to: (a) overlap spatially; (b) overlap temporally (flowering periods); and (c) be sufficiently close biologically that the resulting hybrids are fertile, facilitating introgression of genetic material into a new population. The probability of and extent of gene flow varies according to these limiting factors (Légère, 2005).

Gene flow from transgenic crops to wild relatives may have negative environmental effects if the hybrid plants inherit an increased capacity for invasiveness and weediness of a species (e.g., by conferring a trait such as herbicide tolerance to a specific/related active ingredient would be problematic if that was the only mechanism of eradication) (Andow and Zwahlen, 2006). Furthermore, gene flow from GM plants may be difficult to contain, demonstrated by transgene movement in rice (traits such as high protein content, disease and insect resistance and herbicide and salt tolerance), creeping bentgrass (herbicide tolerance), and oilseed rape (herbicide tolerance) (Rieger et al., 2002; Warwick et al., 2003; Chen et al., 2004; Watrud et al., 2004; Zapiola et al., 2008). This could lead to the evolution of highly competitive weeds and the degeneration of the genetic diversity in indigenous grasses.

This study was conducted to assess the likelihood of gene flow from commercial sugarcane to wild relatives in the sugar production regions of South Africa. Factors such as spatial overlap, proximity, flowering synchrony and pollen viability are prerequisites for hybridization to occur. Therefore, if close relatives occur in areas where sugarcane is cultivated, then transgenic sugarcane presents a likelihood for gene flow to these species. To assess this possibility, the objectives are as follows: (i) review the literature to identify the wild relatives of *Saccharum*, collate what is known about gene flow between cultivated *Saccharum* hybrids and wild relatives in South Africa, determine overlapping flowering times and assess pollen viability of commercial sugarcane; (ii) quantify the distribution of wild *Saccharum* relatives and assess the spatial overlap of their distributions with commercial sugarcane plantations; (iii) determine phylogenetic relationships within the *Saccharum* complex to confirm which species are most closely related to cultivated sugarcane; (iv) make an assessment of the likelihood of gene flow potential between related species and cultivated sugarcane.

## Materials and methods

### Phytogeography of *Saccharum* wild relatives in South Africa

Wild relatives which diverged from *Saccharum* <7.3 million years ago (based on chloroplast sequence chronograms) were identified from a global phylogeny based on chloroplast genomes/regions for the Poaceae (Skendzic et al., 2007; Soreng et al., 2015; Lloyd Evans and Joshi, 2016a). Eleven species of the Sorghinae and Saccharinae subtribes of the Andropogoneae were selected for spatial analyses based on their presence within the sugarcane cultivation region of South Africa: four species that belong to Saccharinae and seven to Sorghinae (Organisation for Economic Cooperation and Development, 2013; Fish et al., 2015; Soreng et al., 2015). Grass nomenclature is in accordance with The Plant List (2013).

Herbarium specimens were sourced from 11 South African herbaria. All specimen data were captured and a gap analysis conducted for the study area to identify where insufficient information was available regarding the occurrence of wild relatives. At these sites, sugarcane field margins were examined for the target species, especially at the preferred habitats of sugarcane relatives such as disturbed and waterlogged areas. Collections were made during flowering periods, May to July, of 2016 and 2017. Field data of collected species were recorded and specimens accessioned in the A. P. Goossens Herbarium (PUC) and National Herbarium (PRE). Herbarium distribution records of the new collections were added to the master database to construct a distribution map per species with ArcGIS (student edition version 10.3, Esri, USA) to confirm their presence in sugarcane cultivation areas (Supplementary Figure 1).

### Plant material

Leaf samples from *Saccharum* hybrid parental breeding lines were collected at SASRI, Mount Edgecombe (23 May 2016). Leaf samples from commercial sugarcane cultivars were collected from grower plantations (4–7 July 2016). Herbarium records and iSpot^2^ were used to pinpoint localities and habitat types where selected wild relatives of *Saccharum* have been collected in the past and are known to occur. Samples of plant leaf material were collected from these locations, for which plant specimens are deposited in the A.P. Goossens Herbarium (PUC) (Table 1). The leaf material was decontaminated with 70% (v/v) ethanol and stored in 50 ml plastic tubes (Thermo Scientific Group) filled with 15 g silica gel. Related species and outgroups that could not be collected in the field were sourced from GenBank genetic sequence database (Table 2).

**Table 1:** Herbarium accession numbers of the different flowering sugarcane cultivars tested for pollen viability and sampled for genomic DNA extraction in 2016 and 2017.

**Sugarcane cultivar**	**GPS Coordinates**	**Location of plantation**	**Herbarium accession no**.
**2016**			
*Saccharum* hybrid cv N36	25°36′08″ S, 31°33′30″ E	Malelane	PUC 14606
*Saccharum* hybrid cv N23	25°30′05″ S, 31°26′09″ E	Malelane	PUC 14609
*Saccharum* hybrid cv N36	25°33′08″ S, 31°56′09″ E	Komatipoort	PUC 14615
*Saccharum* hybrid cv N14	25°33′04″ S, 31°56′02″ E	Komatipoort	PUC 14616
*Saccharum* hybrid cv N28	27°28′05″ S, 32°09′02″ E	Jozini	PUC 14617
*Saccharum* hybrid cv N19	27°28′04″ S, 32°09′02″ E	Jozini	PUC 14620
*Saccharum* hybrid cv N43	27°26′03″ S, 32°10′00″ E	Jozini	PUC 14621
*Saccharum* hybrid cv N25	27°26′03″ S, 32°09′09″ E	Jozini	PUC 14622
*Saccharum* hybrid cv N36	27°24′08″ S, 32°09′05″ E	Jozini	PUC 14626
*Saccharum* hybrid cv N41	27°25′07″ S, 32°10′19″ E	Jozini	PUC 14628
*Saccharum* hybrid cv N23	27°23′08″ S, 31°39′08″ E	Jozini	PUC 14629
*Saccharum* hybrid cv N42	28°43′08″ S, 31°55′01″ E	Empangeni	PUC 14630
*Saccharum* hybrid cv N26	28°43′33″ S, 31°48′41″ E	Empangeni	PUC 14631
*Saccharum* hybrid cv N19	28°44′05″ S, 31°54′05″ E	Empangeni	PUC 14632
*Saccharum* hybrid cv NCo376	29°42′12″ S, 31°02′35″ E	Mount Edgecombe	PUC 14656
**2017**			
*Saccharum* hybrid cv N36	25°36′55″ S, 31°33′12″ E	Malelane	PUC 14678
*Saccharum* hybrid cv N14	25°36′01″ S, 31°33′11″ E	Malelane	PUC 14679
*Saccharum* hybrid cv N23	25°37′28″ S, 31°32′57″ E	Malelane	PUC 14680
*Saccharum* hybrid cv N14	25°33′18″ S, 31°55′51″ E	Komatipoort	PUC 14681
*Saccharum* hybrid cv N36	25°33′40″ S, 31°55′44″ E	Komatipoort	PUC 14682
*Saccharum* hybrid cv N23	27°25′22″ S, 31°38′35″ E	Pongola	PUC 14683
*Saccharum* hybrid cv N14	27°24′23″ S, 31°37′33″ E	Pongola	PUC 14684
*Saccharum* hybrid cv N43	27°29′05″ S, 32°08′51″ E	Jozini	PUC 14685
*Saccharum* hybrid cv N19	27°28′52″ S, 32°09′30″ E	Jozini	PUC 14686
*Saccharum* hybrid cv N23	27°26′19″ S, 32°09′59″ E	Jozini	PUC 14688
*Saccharum* hybrid cv N36	27°26′06″ S, 32°09′55″ E	Jozini	PUC 14689
*Saccharum* hybrid cv N14	27°26′19″ S, 32°09′59″ E	Jozini	PUC 14690
*Saccharum* hybrid cv N23	28°26′52″ S, 32°13′38″ E	Mtubatuba	PUC 14691
*Saccharum* hybrid cv N42	28°28′39″ S, 32°18′32″ E	Mtubatuba	PUC 14693
*Saccharum* hybrid cv N14	28°29′05″ S, 32°16′18″ E	Mtubatuba	PUC 14694
*Saccharum* hybrid cv NCo376	28°29′10″ S, 32°15′30″ E	Mtubatuba	PUC 14696
*Saccharum* hybrid cv N36	28°27′57″ S, 32°18′02″ E	Mtubatuba	PUC 14697
*Saccharum* hybrid cv N42	28°44′51″ S, 31°55′36″ E	Empangeni	PUC 14698
*Saccharum* hybrid cv N42	29°28′59″ S, 31°08′14″ E	Umhlali	PUC 14699
*Saccharum* hybrid cv N27	30°38′01″ S, 30°30′03″ E	Port Shepstone	PUC 14704
*Saccharum* hybrid cv N42	30°37′59″ S, 30°30′06″ E	Port Shepstone	PUC 14705
*Saccharum* hybrid cv N58	30°38′01″ S, 30°30′08″ E	Port Shepstone	PUC 14706
*Saccharum* hybrid cv N36	30°38′02″ S, 30°30′08″ E	Port Shepstone	PUC 14707
*Saccharum* hybrid cv N39	30°38′57″ S, 30°29′25″ E	Port Shepstone	PUC 14710
*Saccharum* hybrid cv NCo376	30°38′53″ S, 30°29′15″ E	Port Shepstone	PUC 14711
*Saccharum* hybrid cv NCo376	29°42′11″ S, 31°02′34″ E	Mount Edgecombe	PUC 14715
*Saccharum* hybrid cv N42	29°42′12″ S, 31°02′36″ E	Mount Edgecombe	PUC 14716

**Table 2:** Taxa used for phylogenetic analyses to determine relatedness.

**Species name**	**Geographical origin**	**Herbarium accession no**.	**Place of collection (province, place)**	**Reclassification**	**Data source or accession number (ITS; *matK; rbcL*)**
*Saccharum robustum*	Papuasia, South-east Asia	PUC 14591	KZN, SASRI nursery	–	–
*Saccharum arundinaceum*	Papuasia, South-east Asia	PUC 14594	KZN, SASRI nursery	*Tripidium arundinaceum* (Retz.) Lloyd Evans	–
*Saccharum* hybrid cv Rowan Green	SASRI, RSA	PUC 14598	KZN, SASRI nursery	–	–
*Saccharum* hybrid cv Co745	SASRI, RSA	PUC 14600	KZN, SASRI nursery	–	–
*Saccharum* hybrid cv N14	SASRI, RSA	PUC 14614	MP, Komatipoort sugarcane plantation	–	–
*Saccharum* hybrid cv N36	SASRI, RSA	PUC 14606	MP, Malelane sugarcane plantation	–	–
*Saccharum* hybrid cv NCo376	SASRI, RSA	PUC 14656	KZN, SASRI germplasm nursery	–	–
*Trachypogon spicatus*	Southern Africa	PUC 14655	NW, Ikageng roadside	–	–
*Sorghum versicolor*	Africa	PUC 10278	NW, Potchefstroom roadside	*Sarga versicolor* (Andersson) Spangler	–
*Ischaemum afrum*	Africa, India	–	–	–	HM347038.1; KU291467.1; KU291467.1
*Miscanthus junceus*	Southern Africa	–	–	*Miscanthidium junceum* (Stapf) Stapf	SRR3968481; SRR396848; SRR396848
*Hemarthria sibirica*	India, Temperate East Asia	–	–	–	KF163639.1; KF163806.1; KF163515.1
*Sorghum timorense*	Asia, Australasia	–		*Sarga timorense* (Kunth) Lloyd Evans	SRR424217; SRR424217; SRR424217
*Sorghum versicolor* 2	Tropical and Subtropical Africa	–		*Sarga versicolor* (Andersson) Spangler	SRR427175; SRR427175; SRR427175
*Miscanthus capensis*	Southern Africa	–	–	*Miscanthidium capense* (Nees) Stapf	BeauSci; BeauSci; BeauSci
*Saccharum narenga*	Temperate and Tropical Asia, Ethiopia	–		*Narenga porphyrocoma* (Hance ex Trimen) Bor	SRR3399436; SRR3399436; SRR3399436
*Saccharum spontaneum* SES234B	North Africa, India, Temperate and Tropical Asia, Papuasia	–		–	SRR486146; LN849912.1; LN849912.1
*Saccharum spontaneum* SES196	North Africa, India, Temperate and Tropical Asia, Papuasia	–		–	SRR2899231; SRR2899231; SRR2899231
*Saccharum sinense* Tekcha	India, China	–		–	SRR2891264; SRR2891264; SRR2891264
*Saccharum* hybrid cv SP80-3280	Brazil	–		–	SRR1774133; SRR1774133; SRR1774133 HM347038.1; KU291467.1; KU291467.1
*Saccharum officinarum* IJ76-514	Papuasia	–		–	SRR528718; LN849913.1; LN849913.1 SRR3968481; SRR396848; SRR396848
*Saccharum robustum* NG57-054	Papuasia	–		–	SRR2899233; SRR2899233; SRR2899233 AF190756.1; LN906656.1; KR737308.1
*Miscanthus sacchariflorus* Hercules	Temperate north-east Asia	–		–	BeauSci; BeauSci; BeauSci
*Miscanthus oligostachyus*	Temperate Asia	–		–	HQ822027.1; BeauSci; BeauSci
*Miscanthus floridulus* US56-0022-03	India, Asia Tropical, Asia Temperate, Melanesia, Papuasia, Polynesia	–		–	SRR486154; SRR486154; SRR486154 DQ005089.1; KU556663.1; KP996860.1
*Miscanthus sinensis* Andante	Caucasus, Indo-China, East Asia, Malesia, Australasia, Continental America (introduced)	–		–	BeauSci; BeauSci; BeauSci AF190756.1; LN906656.1; KR737308.1
*Polytoca digitata*	Africa, Triopical Asia, Temperate Asia, Papuasia, Malesia	–		–	GQ870232.1; KY596178.1; KY596178.1
*Polytrias indica*	Tropical west-central Africa, Temperate Asia, China, Malesia, Papuasia, Australasia, Polynesia, South America, Mesoamerica	–		–	GQ870228.1; —; —
*Microstegium vimineum* 2	Africa west-central Tropical, Caucasus, Temperate Asia, Tropical Asia, Indo-China, Malesia	–	–	–	ERR2040772; ERR2040772; ERR2040772
*Bothriochloa insculpta*	Southeast Europe, Africa, Macaronesia, Arabia, Temperate Asia, Tropical Asia, Australasia	–	–	–	AF190756.1; MF963585.1; MF963222.1
*Andropogon glomeratus* var *scabriglumus*	Americas	–		–	MF964041.1; MF963585.1; MF963222.1
*Andropogon virginicus*	Caucasus, Temperate Asia, Australasia, Continental America	–		–	BeauSci; BeauSci; BeauSci
*Hyparrhenia rufa*	Africa, India, Temperate Asia, Tropical Asia, Malesia, Papuasia, Australasia, Continental America	–	–	–	GQ870187.1; KY596156.1; KY596156.1
*Schizachyrium sanguineum*	Tropical Africa, India. China, Temperate Asia, Malesia, Papuasia, Americas	–	–	–	DQ005070.1; KY596124.1; KY596124.1
*Cymbopogon flexuosus*	Eastern Africa, Temperate Asia, Tropical Asia, Malesia, Papuasia	–		–	SRR2970609; SRR2970609; SRR2970609
*Sorghastrum nutans*	Australasia, North America	–		–	DQ005080.1; KU291482.1; KU291482.1
*Sorghum × drummondii*	Central, Eastern and Southern Europe, Eastern and Southern Africa, Malesia, Asia, Continental America	–		–	SRR998968; SRR998968; SRR998968
*Sorghum arundinaceum* 2	North Africa, Macronesia, Tropical Asia, India, Papuasia, Australasia	–		–	SRR999026; SRR999026; SRR999026
*Sorghum halepense* 2	Central, Southern and Eastern Europe, North Africa, Macronesia, Temperate Asia, Tropical Asia, Malesia, Papuasia, Australasia	–		–	SRR486216; SRR486216; SRR486216 HM347038.1; KU291467.1; KU291467.1
*Sorghum propinquum* 369-1	Temperate Asia, Tropical Asia, Malesia, Papuasia, Pacific	–		–	SRR998982; SRR998982; SRR998982 SRR3968481; SRR396848; SRR396848
*Germainia capitata*	Temperate China, Tropical Indo-China, Australasia, Papuasia	–		–	GQ870198.1; KY596175.1; KY596175.1 AF190756.1; LN906656.1; KR737308.1
*Microstegium japonicum*	Temperate Asia, Caucusus, Eastern Asia	–		–	KF163847.1; KF163826.1; KF163826.1
*Microstegium nudum*	Southern Africa, Tropical Africa, Temperate Asia, Tropical Asia, Malesia, Papuasia, Australasia	–		–	EU489073.1; —; MF998299.1
*Saccharum arundinaceum* 2	Temperate Asia, Tropical Asia, India, Malesia, Papuasia	–		*Tripidium arundinaceum* (Retz.) Lloyd Evans	BeauSci; SASRI; SASRI DQ005089.1; KU556663.1; KP996860.1
*Saccharum ravennae*	Temperate Asia, Tropical Asia, India, Malesia, Papuasia	–		*Tripidium ravennae* (L.) H. Scholz	[AF019824.1/AY116296.1]; SASRI; SASRI DQ005089.1; KU556663.1; KP996860.1
*Imperata cylindrica*	Southwestern Europe, North Africa, East Africa, Southern Africa, Temperate Asia, Tropical Azsia, Malesia, Papuasia, Australasia	–			SRR4280862; SRR4280862; SRR4280862 AF190756.1; LN906656.1; KR737308.1
*Tripsacum dactyloides*	Mesoamerica, Caribbean, Australasia	–		–	SRR5127199; SRR5127199; SRR5127199
*Zea mays* B73	Mesoamerica	–		–	SRR447986; KF241981.1; KF241981.1
*Sorghum bicolor BTx623*	North, East and West African in origin, globally distrubuted	–		–	SRR3923525; EF115542.1; EF115542.1 DQ005089.1; KU556663.1; KP996860.1
*Sorghum laxiflorum*	Tropical Asia, Malesia, Papuasia, Australasia	–		–	AF019824.1; —; — DQ005089.1; KU556663.1; KP996860.1
*Cleistachne sorghoides*	Tropical and Subtropical Africa, Arabia, Tropical Asia, Temperate Asia	–		–	U04790.1; —; —

*For data sources, all entries beginning with SRR or ERR were downloaded from NCBI's sequence read archive and sequences were assembled as SASRI. Sequences labeled BeauSci or SASRI were either gifted by BeauSci Ltd. or were within the SASRI short read collection. Unlabelled sequences were collected and sequenced at SASRI. All other sequences were downloaded from the NCBI nucleotide archive. The symbol — in the accession no. column indicates that sequence information was not available. Where there are two GenBank accessions for a sequence, this indicates that these sequences were merged prior to analysis—shown in []. NW—North West Province, RSA; KZN—KwaZulu-Natal Province, RSA; SASRI—South African Sugarcane Research Institute, Mount Edgecombe, KZN, RSA*.

### DNA extraction, amplification, and sequencing

Between 0.10–0.15 g of dry plant leaf material per species was homogenized in liquid nitrogen and genomic DNA was isolated (GeneJET Plant Genomic DNA Purification kit; Thermo Fisher Scientific, USA) according to the manufacturer's protocol. The purity and concentration of the DNA was assessed (NanoDrop ND-1000 spectrophotometer; NanoDrop Technologies, Inc., Thermo Scientific Group).

DNA sequences of the internal transcribed spacer (ITS) regions of the 5.8s ribosomal gene as well as that of two chloroplast genes, ribulose-bisphosphate carboxylase (*rbcL*) and maturase K (*matK*) were used to design primers (Table 3). Amplification of the above three regions was done via Polymerase Chain Reaction (PCR) on a C1000 Thermal Cycler (BioRad, USA). The reaction mixture included 2X KAPA Taq readyMix PCR kit (1x PCR buffer, 2 U Taq DNA polymerase, 0.2 mM of each DNTP, 1.5 mM MgCl_2_ and stabilizers), 0.5 μM forward and reverse specific primers, 5–50 ng DNA template and nuclease-free water. For each primer set (Table 3) the initial denaturation step was at 94°C for 3 min, followed by denaturation at 94°C for 60 s. Annealing temperatures varied depending on the primer set: 50°C for 30 s (ITS and *rbcL*) and 48°C for 40 s for *matK*.; the extension step was at 72°C for 30 s (ITS and *rbcL*) and 60 s for *matK*. There were 35 thermocycles for ITS and *rbcL* and 40 for *matK*. The final extension step was at 72°C for 10 min. PCR products were visualized on a 1% (w/v) agarose gel and cleaned-up (GeneJET PCR purification kit; Thermo Fisher Scientific, USA).

**Table 3:** The primers used for the amplification and sequencing of the internal transcribed spacer (ITS), ribulose-bisphosphate carboxylase (*rbcL*), and maturase K (*matK*) gene fragments used as the basis for the phylogenetic analyses.

**Primer name**	**5′-3′ primer sequence**	**Size (bp)**	**References**
*rbcL*a-F	ATG TCA CCA CAA ACA GAG ACT AAA GC	±550	Kress et al., 2009
*rbcL*a-R	GTA AAA TCA AGT CCA CCR CG		
ITS 4F	TCC TCC GCT TAT TGA TAT GC	±650	White et al., 1990; Stanford et al., 2000
ITS 5A	CTT TAT CAT TTA GAG GAA GGA G		
*matK* 472F	CCC RTY CAT CTG GAA ATC TTG GTT	±750	Yu et al., 2011
*matK* 2148R	GCT RTR ATA ATG AGA AAG ATT TCT GC		

*F, forward; R, reverse*.

Sequencing reactions were performed with the same primers as those used for PCR using the BigDye Terminator V1.3 cycle sequencing kit (Applied Biosystems, USA). This was followed by fluorescence-based DNA analysis using capillary electrophoresis technology on the Applied Biosystems 3500 Genetic Analyser. Sequences were analyzed and trimmed using Sequencing Analysis V5.3.1 (Applied Biosystems).

### Sequence assembly

The 5.8s genomic ITS cassette along with the chloroplastic *matK* and *rbcL* genes were chosen for phylogenetic analysis. In those cases where no ITS, *matK*, or *rbcL* sequences could be found in GenBank, sequences were assembled from short read data (either mined from NCBI's SRA archive^3^ or made available through on-going collaborations) (Table 2) using a bait-and-assemble assembly method described previously (Lloyd Evans and Joshi, 2016b). Third party data assembled for this study are noted in Table 2 and the assemblies are provided as Supplementary File 1.

### Sequence alignments

The ITS cassette (18s rRNA partial, ITS1 complete, 5.8s rRNA, ITS2 complete, 28s rRNA partial) region was aligned as described previously (Martin et al., 2017). Briefly, DNA sequences (Table 2) were aligned with SATÉ (Liu et al., 2009) using MAFFT (Katoh and Standley, 2013) as the aligner, MUSCLE (Edgar, 2004) as the sub-alignment joiner and RAxML as the tree estimator. The final RAxML tree was used as input for PRANK (Löytynoja et al., 2012) an indel-aware alignment optimizer. PRANK was run for 5 generations, using RAxML (identifying the most likely tree from 100 samples) for Maximum Likelihood (ML) tree estimation until both the alignment and the tree topology stabilized. The chloroplastic *matK* and *rbcL* sequences were aligned with SATÉ.

Long-branch attraction and incomplete sampling (Philippe et al., 2017) can be major confounding effects in phylogenetic inference. In an attempt to minimize these effects, at least two exemplars for each sequence were included in the initial alignment and as many species and genera were sampled as possible. To test for long-branch attraction a custom PERL script was written. This script removed one sequence at a time from the final alignment. The reduced alignment was analyzed with RAxML where the most likely tree was identified from 100 random replicates. After the analysis, all trees were compared and where the initial reference tree and the resampled tree differed significantly the deleted sequence was labeled as responsible for long-branch effects and was removed from all subsequent analyses. The sequences remaining after this test were re-aligned using SATÉ and PRANK, as described above. These sequences yielded the final alignment. The final ITS alignment and phylogeny along with the *matK* alignment and phylogeny and the *rbcL* alignment and phylogeny were deposited in TreeBase^4^.

Wherever possible, the entire ITS cassette was used. However, where no alternate data was available, the shorter assemblies from existing sequence data were integrated into the alignment and padded with Ns.

### Partition analyses

The ITS cassette was divided into 18s rRNA, ITS1, 5.8s rRNA, ITS2, and 28s rRNA regions, whilst the entire *matK* and *rbcL* genes were analyzed as a single partition. Best-fit evolutionary models were determined using jModelTest2 (Darriba et al., 2012) under the AICc criterion. The best fit models were found to be: 18s RNA: TVM + G; ITS1: TPM3uf + G; 5.8s rRNA: JC + G; ITS2: GTR + G; 28s rRNA: GTR + G; *matK*: TVM + G; and *rbcL*: HKY + I.

### Phylogenetic analyses

Phylogenetic analyses were run for the ITS cassette along with separate analyses for *matK* and *rbcL*. Non-parametric bootstrap tests (using the above partitioning schema) and SH-aLRT analyses were run with IQ-Tree (Nguyen et al., 2015). Neighbor-Joining analyses were run with APE (Paradis et al., 2004). Bayesian Inference (BI; again using the above partitioning schema) was run with MrBayes (Ronquist and Huelsenbeck, 2003). IQ-Tree analyses were run for 2,000 replicates. MrBayes analyses were run with 50,000,000 generations with sampling every 100th tree. Two independent MrBayes analyses, each of two independent runs, were conducted. To avoid any potential over-partitioning of the data, the posterior distributions and associated parameter variables were monitored for each partition using Tracer v 1.6 (Rambaut et al., 2017). High variance and low effective sample sizes were used as signatures of over-sampling. Burn-in was determined by topological convergence and was judged to be sufficient when the average standard deviation of split frequencies was <0.002 along with the use of the Cumulative and Compare functions of AWTY (Nylander et al., 2008). The first 30% of sampled trees were discarded as burn-in.

Phylogenetic analyses (ML and BI) were summarized with Sumtrees (Sukumaran and Holder, 2010) prior to drawing with FigTree (2017) and finishing with Adobe Illustrator to generate publication-quality figures. The ITS only, *matK*, only and *rbcL* only tree topologies were deposited in TreeBase^4^.

### Chronogram generation with r8s

The application r8s (Sanderson, 2003) was employed for chronogram generation. An optimal tree topology was generated and was used for analysis. Parameters were adjusted for ML branch lengths on all trees and divergence timings were estimated with a smoothing factor of 100, the Penalized Likelihood method using the Truncated Newton optimization framework with analytical gradients generated by r8s. To generate 95% confidence intervals on branch times, the non-parametric bootstrap trees generated by IQ-Tree were used as input to r8s. All trees were concatenated into a single nexus file using a custom PERL script and an r8s block was appended so that r8s could be executed over all trees with parameters as defined above. The *profile* command of r8s was employed to individually summarize the distribution of ages at all given nodes of the tree (employing a custom PERL wrapper). Priors for the main nodes were defined as follows: root, fixed age of 13.8 million years ago, *Tripsacum–Germainia* node, fixed age of 9.2 million years ago (Estep et al., 2014), *Sarga–Miscanthidium* node, minimum age of 7.4 million years ago, *Miscanthus–Miscanthidium* node fixed age 3.4 million years ago, *S. spontaneum*–*S. sinense* node, minimum age of 1.4 million years ago (Lloyd Evans and Joshi, 2016a). All other nodes were unconstrained.

### Pollen viability testing

**Figure 1:**
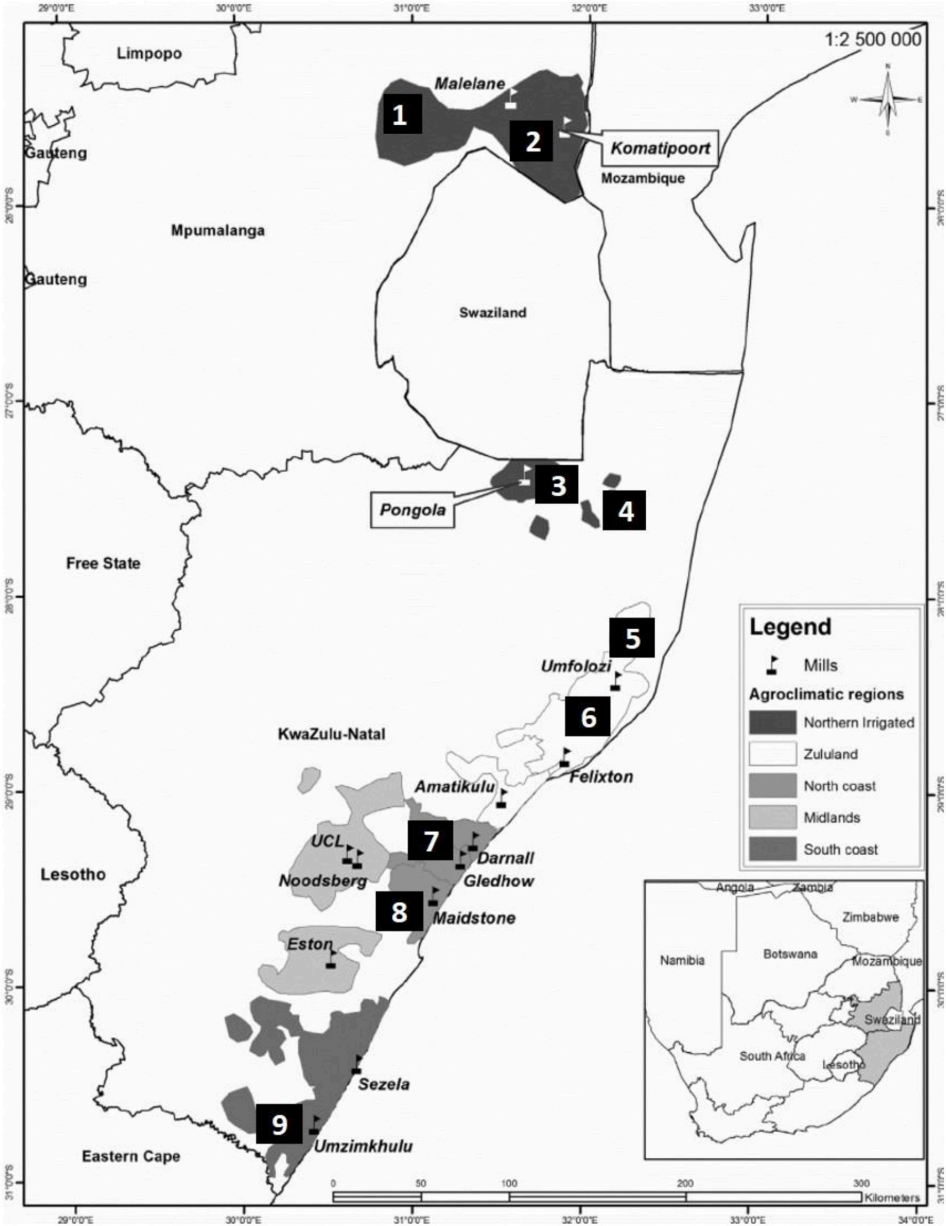
Sugarcane production regions and locations of sugar mills in the Mpumalanga and KwaZulu-Natal provinces of South Africa. Sites for pollen collection were as follows: 1: Malelane; 2: Komatipoort (Mpumalanga); 3: Pongola; 4: Jozini; 5: Mtubatuba; 6: Empangeni; 7: Umhlali; 8: Mount Edgecombe; and 9: Port Shepstone (KwaZulu-Natal).

Pollen samples from commercial sugarcane cultivars were collected during the flowering season (July 2016 and 2017) from nine different sites in South Africa, two in Mpumalanga and seven in KwaZulu-Natal (Figure 1). Sites 1–5 are situated in the irrigated region while sites 6–9 are rain-fed. Fresh pollen was collected from anthers in dehiscence, from three separate inflorescences per cultivar per site. Inflorescence collection was between 6.00 and 8.30 h and viability tests conducted in the field immediately thereafter (Amaral et al., 2013).

Two stains were used to estimate pollen viability: 2,3,5-triphenyl tetrazolium chloride (TTC) (Soares et al., 2013) and iodine potassium iodide (IKI) (Huang et al., 2004). Pollen grains were stained with IKI [1% (w/v) iodine and 2% (w/v) potassium iodide in distilled water] for 5 min, while those stained with TTC [1% (w/v) TTC and 5% (w/v) sucrose in distilled water] were examined after 15 min of incubation in direct sunlight. Viewing was under a compound microscope (Model 11, Wild, Heerbrugg Switzerland) at 100 × magnification and counting was aided using a grid stuck to the underside of each glass slide. A random count of a minimum of 100–150 pollen grains was performed for each cultivar replicate, and the percentage viability was determined as the ratio of viable pollen grains (intense dark color for IKI and deep pink for TTC) divided by the total number of grains.

An average from three pollen counts per cultivar per locality was used for calculating percentage pollen viability. All statistical analyses were carried out using Statistica (version 13; Dell Inc., USA). The Kolmogorov-Smirnoff and Lilliefors tests for normality showed that the data did not meet the assumptions of normality in the distribution of all variables. Therefore the Kruskal-Wallis analysis of variance (ANOVA; non-parametric statistics) for comparing multiple independent groups was used to determine differences between determinants measured.

Environmental data including relative humidity, soil water content at 100 mm depth, minimum and maximum temperatures were extracted from the SASRI weather web^5^. Automatic weather stations were situated at each of the sampling sites. Data was extracted from the first of May 2016 and 2017 up to the day at which sampling took place for each of the sites. Mean values were used for each environmental variable at each site. Day length data with the same time resolution and period was obtained online^6^. The non-parametric Spearman rank correlation coefficient was calculated as a measure of correlation between all possible pairs of variables and significance was tested at the 0.05 level.

### Desk-top study of hybridization

Prominent literature was consulted to assess gene flow potential. Printed evidence of reproductive compatibility and the formation of hybrids between commercial sugarcane with target related species were used to assess the likelihood of hybridization. The numbers of publications which reported hybridization were recorded. Successes were scored if the publications reported formation of hybrid progeny (FitzJohn et al., 2007; McGeoch et al., 2009; Organisation for Economic Cooperation and Development, 2013) and ranked accordingly. In cases where literature recorded hybridization evidence between *Saccharum* hybrids and wild relatives, the following approaches were undertaken: (i) if target species were reported to hybridize with *Saccharum* hybrids, the number of publications and successes were recorded and scored 1 per event; (ii) if species not found in South Africa hybridized with *Saccharum* hybrids, and the genus is present in the sugar production area, the species from such genera were treated as reproductively compatible with commercial sugarcane and the number of publications and successes recorded and scored 0.5 per event. The wild relative-*Saccharum* crosses with most hybrids ranked the highest and species with fewer hybrids were ranked lower.

### Flowering times

Flowering times were assessed using literature, herbarium specimens and collections made during field surveys. *Saccharum* hybrids flower from March to August in South Africa (Sithole and Singels, 2013; Zhou, 2013). Plant specimens with inflorescences, dates of collections and occurrence in the study area were used to analyse flowering times in addition to collections sampled during the study. The overlapping percentages between the flowering time of *Saccharum* hybrids and each wild relative was calculated by dividing the number of overlapping months with the total number of months of sugarcane flowering. The wild relatives with more overlapping months were ranked the highest and species with less overlap were ranked lower.

### Spatial assessment

The qualitative assessment to determine the likelihood of wild relatives co-occurring with cultivated sugarcane, which may enhance gene flow potential, was based on the following factors: prevalence, spatial overlap, proximity, distribution potential, gene flow potential, and flowering times (Ellstrand et al., 1999; Chapman and Burke, 2006; Schmidt and Bothma, 2006; Tesso et al., 2008; McGeoch et al., 2009; Andriessen, 2015). All target species were assessed and ranked per factor, whereby species with highest rank was scored 11 and species with lowest rank was scored 1. In the cases where no information was available for a species, the species could not be ranked and was scored 0 (no evidence equates to no ranking). It would be inaccurate to rank species without data, as it would inflate the likelihood scores for the areas where these species were found.

Sugarcane production areas for Limpopo, Mpumalanga and KwaZulu-Natal were obtained from the 2015 National Land Cover dataset. These areas were then overlaid with a grid of quarter-degree squares (QDS) using ArcGIS to provide 113 mapping units for the spatial assessment (Robertson and Barker, 2006). Some of these QDS overlap with Mozambique and Swaziland, but no data was available for these areas. It should be noted that wild relatives may be present in those jurisdictions and did not form part of this study.

The presence of wild relatives in QDS of sugarcane cultivation areas were used to calculate their prevalence, i.e., how common these species are in the study area. The number of individuals per species per QDS within the sugarcane cultivation area was determined. The proportion of individuals per species within QDS was calculated. The same procedure was followed for QDS bordering sugarcane cultivation areas. These proportions were summed to determine the proportional prevalence of each species in the study area. These prevalence values were then sorted from highest to lowest proportion of individuals per species within and bordering sugarcane QDS and scored.

Spatial overlap is the notion of similarity in distribution patterns (or shared occurrences). It was calculated for each species by dividing the number of QDS that overlap with sugarcane cultivation areas with the total number of QDS for sugarcane cultivation areas. This derived a percentage of overlap per species. Species were ranked from highest to lowest based on overlap percentage, with the highest rank scoring 11 and lowest rank scoring 1.

Pollen of graminoids can travel up to 700 m from the donor plant (Schmidt and Bothma, 2006). This was set as the cut-off for proximity measures both during field work and extracting data from herbarium specimens. The herbarium record database was used to construct a table of habitat notes per species and the presence or absence of wild relatives in the vicinity of sugarcane fields were noted. These records were combined with confirmations from the literature and field surveys. Species with more occurrences within the 700 m zone (high proximity) were ranked higher than species with few or no records in sugarcane fields and margins.

Weedy grasses are often spread by different modes of transport (Milton, 2004). Transport networks therefore gives an indication of the potential for weedy relatives of sugarcane to spread, with denser networks implying higher chances for migrations. Road and railway networks were used to calculate the spatial distribution potential of wild relatives across the study area. For each species the number of railway lines and roads per QDS were counted respectively. Totals of QDS containing railways and roads per species were summed. Higher totals were considered indicative of a wild relative's ability to disperse and ranked as highest likelihood for the species to spread to sugarcane fields (Knispel et al., 2008).

### Likelihood scores

Likelihood scores were calculated per species to determine which *Saccharum* relatives might present a higher likelihood for gene flow with sugarcane based on relatedness, flowering time and spatial assessment. Factors were weighted equally for relatedness and spatial assessments (Butler et al., 2007). Relatedness was calculated from the phylogenetic classification and hybridization events, and spatial assessment involved prevalence, spatial overlap, proximity, and distribution potential. Thereafter, spatial, temporal (flowering time) and relatedness assessments were weighted 1:1:2 to come up with a final likelihood score. This weighting was based on the assumption that gene flow and relatedness are not correlated due to reproductive barriers such as flowering time (Panova et al., 2006), and that gene flow likelihood is evenly dependent on temporal and spatial assessment factors. Relatedness is weighted more as it becomes the determining factor for gene flow when prevalence, spatial overlap, proximity, distribution potential or flowering time provide the required compatibility for pollen from one species to reach the stigma of another species.

Likelihood maps indicating various levels of potential for gene flow to occur between *Saccharum* hybrids and wild relatives within sugarcane production areas of eastern South Africa was constructed based on the factor scores per species and summed per grid. The following classes were used for assessing the likelihood for gene flow: *Sorghastrum nudipes* scored 6 and there was no sugarcane QDS containing only this wild relative species. QDS with sugarcane plantations without wild relatives (0–12); sugarcane QDS plantations with wild relatives: very low (13–43); low (44–86); high (87–129); very high (130–172).

## Results

### Assessing hybridization potential from the literature

A literature review of hybridization events between cultivated sugarcane and its relatives, revealed 39 hybridization incidents were reported in 23 different studies dating from 1935 to 2014 (reviews by Bourne, 1935; Gao et al., 2014). From these, there were only three claims of spontaneous hybridization (Parthasarathy, 1948; Ellstrand et al., 1999), with the remaining crosses requiring human intervention in artificially controlled conditions using experimental procedures that maximized flowering, pollination and seedling survival. Crosses were performed to integrate the beneficial traits of one species to another to enhance agronomic traits such as growth, ratoonability and biomass accumulation (Brett, 1950; Piperidis et al., 2000; Aitken et al., 2007; Gao et al., 2014).

The genus previously known as *Erianthus* (now divided into *Tripidium* and *Saccharum*) was utilized in 18 of the artificial man-made crosses, predominantly with *Saccharum arundinaceum* (synonym *Erianthus arundinaceus, Tripidium arundinaceum*). Similarly, the number of crosses made with cultivated sugarcane was mainly with the *Saccharum* genus (10 crosses) and with *S. arundinaceum* (4 crosses). Other genera which have been crossed with sugarcane include *Bambusa, Imperata, Miscanthidium, Sorghum*, and *Zea*. Of the 18 species that have been involved in hydridization with sugarcane, seven occur in South Africa and comprise 30.77% of the total hybridization events. The highest number of seedling survival in cultivation was 1,371, resulting from *Saccharum* hybrids × *Sorghum bicolor* (L.) Moench, representing a 9.7% recovery rate from 14,141 total seedlings produced from the crosses (Hodnett et al., 2010). The lowest seedling survival was from a cross involving *Zea mays* L., where only one from more than 1,000 seedlings survived (Bonnett et al., 2008). One of the reported crosses involving *S. bicolor* failed with no true seedlings obtained (Bourne, 1935). With the exclusion of the former attempt, 48.72% studies used molecular markers to verify the presence of the maternal and paternal alleles from putative hybrids, whereas the remaining crosses (51.38%) relied on visual inspection of inherited morphological characteristics against those of parent lines as well as chromosome counts (Khanyi, 2018).

*Imperata cylindrica, Sorghum arundinaceum, S*. × *drummondii*, and *S. halepense* were the only species that were found to be reproductively compatible with *Saccharum* species based on assessed literature (Table 4). *Miscanthidium capense* and *Miscanthidium junceum* were not part of any species-specific hybridization studies, but were scored as compatible reproductive species based on the literature reporting on other species of the genus hybridizing with *Saccharum* species (Table 4). *Miscanthidium* hybridization is especially documented in the literature (17 publications) of which six reported successes. Hybridization potential between *Miscanthidium* and *Saccharum* ranked highest, *I. cylindrica* was reported in five publications with one success and *S. halepense* was recorded in two publications with one success (Table 4). There were considerably more publications on other *Sorghum* species hybridizing with *Saccharum* species, which was not included in the analyses due to uncertainty regarding the generic divisions within the *Sorghum* complex.

**Table 4:** Summary of gene flow reports between *Saccharum* hybrids and wild relatives from the literature for genera present in the sugarcane cultivation areas.

**Species**	**No. publications reporting hybridization**	**No. reports of successful hybridization**	**Success %**	**Score**
*Cleistachne sorghoides*	–	–	0	0
*Imperata cylindrica*	5	1	20	7
*Microstegium nudum*	–	–	0	0
*Miscanthidium* spp.	9	3	33	8
*Sarga versicolor*	–	–	0	0
*Sorghastrum nudipes*	–	–	0	0
*Sorghastrum stipoides*	–	–	0	0
*Sorghum arundinaceum*	1	1	100	11
*Sorghum* × *drummondii*	1	1	100	11
*Sorghum halepense*	2	1	50	9

*Rankings were based on the number of successful hybridization events, with the highest ranking scoring 11. A score of 0 was given when no instances of hybridization were reported in the literature and therefore no gene flow risk is currently known (no evidence equates to no ranking). Miscanthidium was treated at species level as hybridization was not conducted with species found in South Africa*.

### Occurrence of andropogoneae in sugarcane cultivation areas

A total of 815 herbarium specimens of 11 *Saccharum* wild relative species were sourced from 11 herbaria. These records were supplemented by 34 observations of *Saccharum* wild relatives during field visits to sugarcane cultivation areas in South Africa. All 11 wild relatives of the Andropogoneae have been recorded from sugarcane cultivation areas. Six species occurred throughout the sugar cultivation region, but *M. capense* (previously *Miscanthus capensis*), *Sorghum* × *drummondii*, and *Sorghastrum stipoides* were restricted to the southern parts, and *Cleistachne sorghoides*, and *S. nudipes* to the northern parts of the cultivation area.

### Pollen viability of commercial sugarcane cultivars

**Figure 2:**
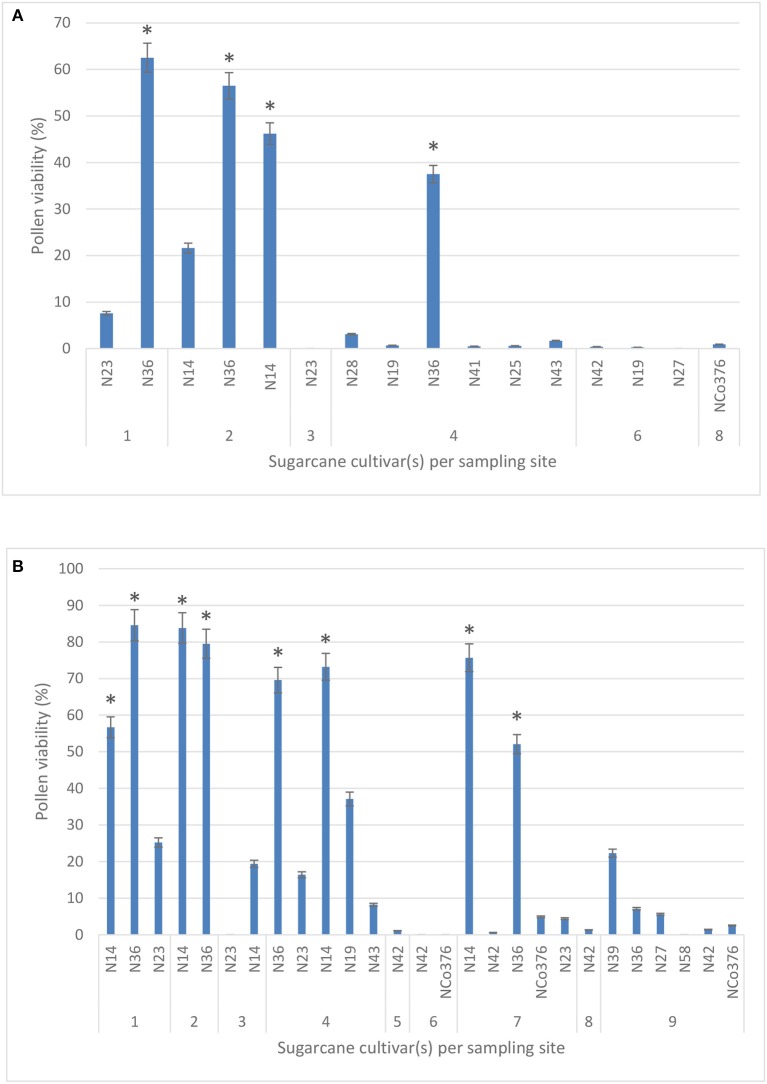
Pollen viability (%) of 11 sugarcane cultivars measured across six study sites using the 2,3,5-triphenyl tetrazolium chloride stain in 2016 **(A)** and 2017 **(B)**. Sites −1: Malelane; 2: Komatipoort (Mpumalanga); 3: Pongola; 4: Jozini; 5: Mtubatuba; 6: Empangeni; 7: Umhlali; 8: Mount Edgecombe; and 9: Port Shepstone (KwaZulu-Natal). Values are means ± SE (*n* = 3). Significant differences are indicated by a ^*^ (ANOVA).

A total of 11 sugarcane cultivars were tested for pollen viability during 2016 from six sites in the study area. Pollen viability tests during 2017 included two additional cultivars, N39 and N58, from site 9. No significant difference in pollen viability using two stains, IKI (40.5%) and TCC (38.1%), was observed when comparing 42 individual counts (Kruskal-Wallis ANOVA; *p* = 0.622), therefore results presented are those obtained using the TTC stain for 2016 and 2017 (Figures 2A,B, respectively).

For both years, 2016 and 2017, the highest mean percentage viability was observed in cultivar N36 (62.5 and 84.6%, respectively), followed by N14 (46.2 and 83.8%, respectively) in the northern irrigated regions of Mpumalanga. Pollen from all the other cultivars (N19, N23, N25, N27, N28, N41, N42, N43, and NCo376) during the same year had lower mean percentages of viability ranging from 0 to 7.6%, while pollen from N23, N42, N58, and NCo376 was not viable in 2017. In 2017, pollen viability decreased from 84.6% in the northern irrigated regions (site 1) to 0% in the southern rain-fed coastal regions of the study area (site 9) (Figure 2), likely due to less favorable environmental conditions. None of the sites had optimal conditions required for flowering (reviewed by Cheavegatti-Gianotto et al., 2011; Organisation for Economic Cooperation and Development, 2013), but percentage pollen viability had a significant positive correlation with both mean maximum temperature (*r* = 0.6) and day length (*r* = 0.5), and a significant negative correlation with soil water content (*r* = −0.4) (results not shown). It must be noted that different cultivars were planted at the sampling sites.

### Flowering times

Information sourced from herbarium labels and field surveys highlighted that *I. cylindrica* and *S. arundinaceum* flower throughout the year, suggesting a 100% flowering synchrony with *Saccharum* hybrids (Table 5). *Miscanthidium capense* has an 83% overlap in flowering time with *Saccharum* hybrids. More than 66% of flowering synchrony was further depicted for *Microstegium nudum, M. junceum, S*. × *drummondii*, and *S. halepense* (Table 5).

**Table 5:** Flowering times of *Saccharum* wild relatives (based on literature, herbarium specimens, and field observations) in sugarcane cultivation areas.

**Species**	**Flowering period (literature)**	**Flowering period (herbarium records and field observations)**	**Combined flowering period**	**No. months with flowering synchrony**	**Overlapping months (%)**	**Score**
*Cleistachne sorghoides*	Feb–Apr	Mar–Apr	Feb–Apr	2	33	2
*Imperata cylindrica*	Aug–Jun	Jan–Dec	Jan–Dec	6	100	11
*Microstegium nudum*	Jan–May	Jan–Jun	Jan–Jun	4	67	7
*Miscanthidium capense*	Nov–Apr	Dec–Jul, Sep	Sep–Jul	5	83	9
*Miscanthidium junceum*	Nov–Jun	Nov–Jun, Sep	Sep–Jun	4	67	7
*Sarga versicolor*	Dec–May	Jan–May	Dec–May	3	50	3
*Sorghastrum nudipes*	Jan–Apr	Jan–Feb, Apr	Jan–Apr	2	33	2
*Sorghastrum stipoides*	Dec–Apr	Nov–May, Aug	Aug–May	4	67	7
*Sorghum arundinaceum*	Jan–Jun	Jan–Dec	Jan–Dec	6	100	11
*Sorghum* × *drummondii*	Jan–Jun	Jan–Mar, Jun–Jul, Nov	Nov–Jul	5	83	9
*Sorghum halepense*	Dec–May	Nov–Mar, May, Jul–Sep	Nov–Sep	4	67	7

*Calculation of scores was based on ranking the percentage flowering synchrony with Saccharum hybrids (flowering from March to August in South Africa). Saccharum wild relative species were ranked from highest to lowest, with highest overlap scoring 11 and lowest 1*.

### Determining genetic relatedness using phylogenetic analyses

**Figure 3:**
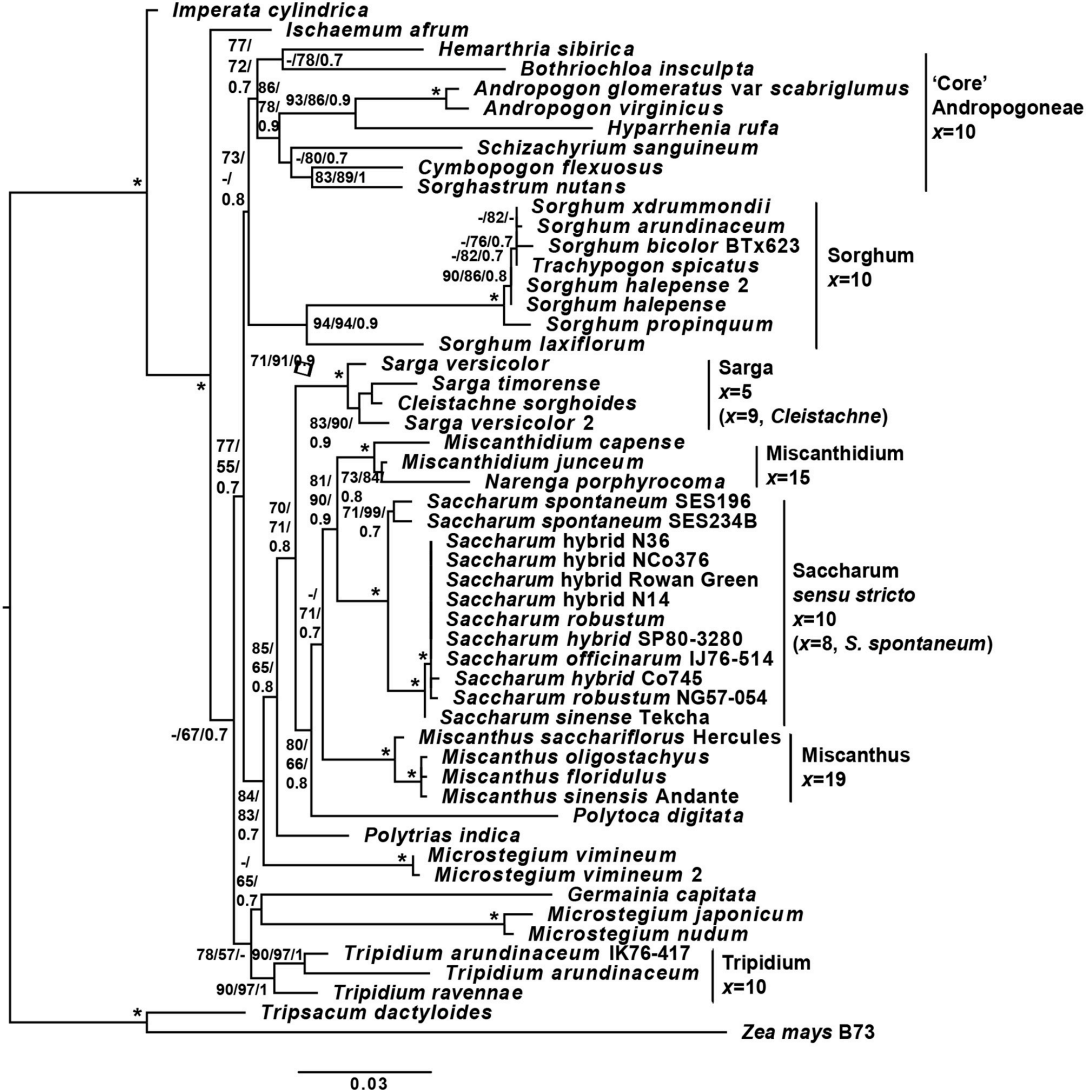
Phylogeny of sugarcane and related genera, based on the ITS cassette. A phylogeny of *Saccharum, Sorghum* and related genera based on the ITS (18s rRNA partial, ITS1 complete, 5.8s rRNA complete, ITS2 complete and 28s rRNA partial) genomic cassette. Tree terminals are the species name and cultivar or accession, where appropriate. Numbers at nodes represent SH-aLRT/non-parametric bootstrap/Bayesian inference support values. Bars to the right of the tree represent major clades, with associated base or monoploid (*x*) chromosome numbers. Branch lengths (scale on the bottom) correspond to the expected numbers of substitutions per sides. Monoploid chromosome numbers are derived from: *Sorghum* and *Sarga*—Gu et al. (1984); *Miscanthus*—Adati (1958); *Miscanthidium*—Strydom et al. (2000); *Saccharum spontaneum*—Ha et al. (1999); *Saccharum officinarum*—Li et al. (1959); *Tripidium*—Jagathesan and Devi (1969); and *Cleistachne*—Celarier (1958). The code ^*^represents complete support for a node (100% SH-aLRT, 100% non-parametric boostrap and Bayesian inference of 1), whilst—represents support that is below the threshold (65% for SH-aLRT, 50% for non-parametric bootstrap and 0.7 for Bayesian inference). Within *Saccharum sensu stricto*, between the sister relationship of *Saccharum robustum* NG57-054, *Saccharum* hybrid cv Co745 and *Saccharum officinarum* IJ76-514 with the remaining species there was insufficient sequence divergence within the ITS cassette to yield any meaningful branch supports between the species. The Tripsacinae (*Tripsacum dactyoides* and *Zea mays*) were employed as an outgroup.

The initial experimental design was based on chloroplast phylogenies. However, during the course of the study, the paper of Folk et al. (2017) highlighted the importance of ancient reticulate evolution and parallel organellar capture in plant evolution. As a result of that paper, we performed an ITS-based phylogeny to check for reticulate evolution in the Andropogoneae. The overall ITS cassette phylogeny (Figure 3) is consistent with previous genomic studies of the Andropogoneae (Estep et al., 2014; Welker et al., 2015). However, we have increased resolution of the core Saccharinae and from our analyses, *Saccharum sensu stricto* (*Saccharum spontaneum* and its sister group) is sister to *Miscanthidium* and *Narenga* with good support. This crown group is in turn sister to *Miscanthus* (with moderate support). The entire grouping is, in turn, sister to *Sarga* (with moderate support).

In common with the findings of Hodkinson et al. (2002) we also see *Polytoca digitata* within this grouping. *Microstegium* is clearly not monophyletic and we place *Microstegium vimineum* (with good support) as an outgroup to the entire clade that might be described as the “Saccharinae.” The core Andropogoneae is sister to the Saccharinae and *Sorghum* is placed as sister to the core Andropogoneae, although with only moderate support (73% SH-aLRT and 0.8 BI). Though the support for the placement of *Sorghum* is not strong, all independent tree topologies (SH-aLRT, maximum likelihood and Bayesian inference) agree on the topology and our placement of Sorghum as sister to the core Andropogoneae is consistent with the work of Hawkins et al. (2015) who analyzed multiple genes. This confirms the presence of reticulate evolution in the origins of Andropogoneae and casts doubt on many conclusions determined from chloroplast only datasets.

Of the two chloroplastic genes chosen for this study, *matK* provided only a relatively weak phylogenetic signal with over 50% of sequences undetermined and *rbcL* provided no phylogenetic signal (data submitted to TreeBase). Both chloroplastic genes failed IQ-Tree statistical testing for phylogenetic signal. Moreover, as the chloroplastic signal for many of the genera (particularly *Imperata* and *Sorghum*) differ (compare: Estep et al., 2014; Hawkins et al., 2015 and Burke et al., 2016) combining genomic (ITS) and chloroplastic (*matK* and *rbcL*) data would be detrimental to the overall topology of the phylogeny, particularly as genomic data is currently considered to present the “true” evolutionary signal (Estep et al., 2014).

**Figure 4:**
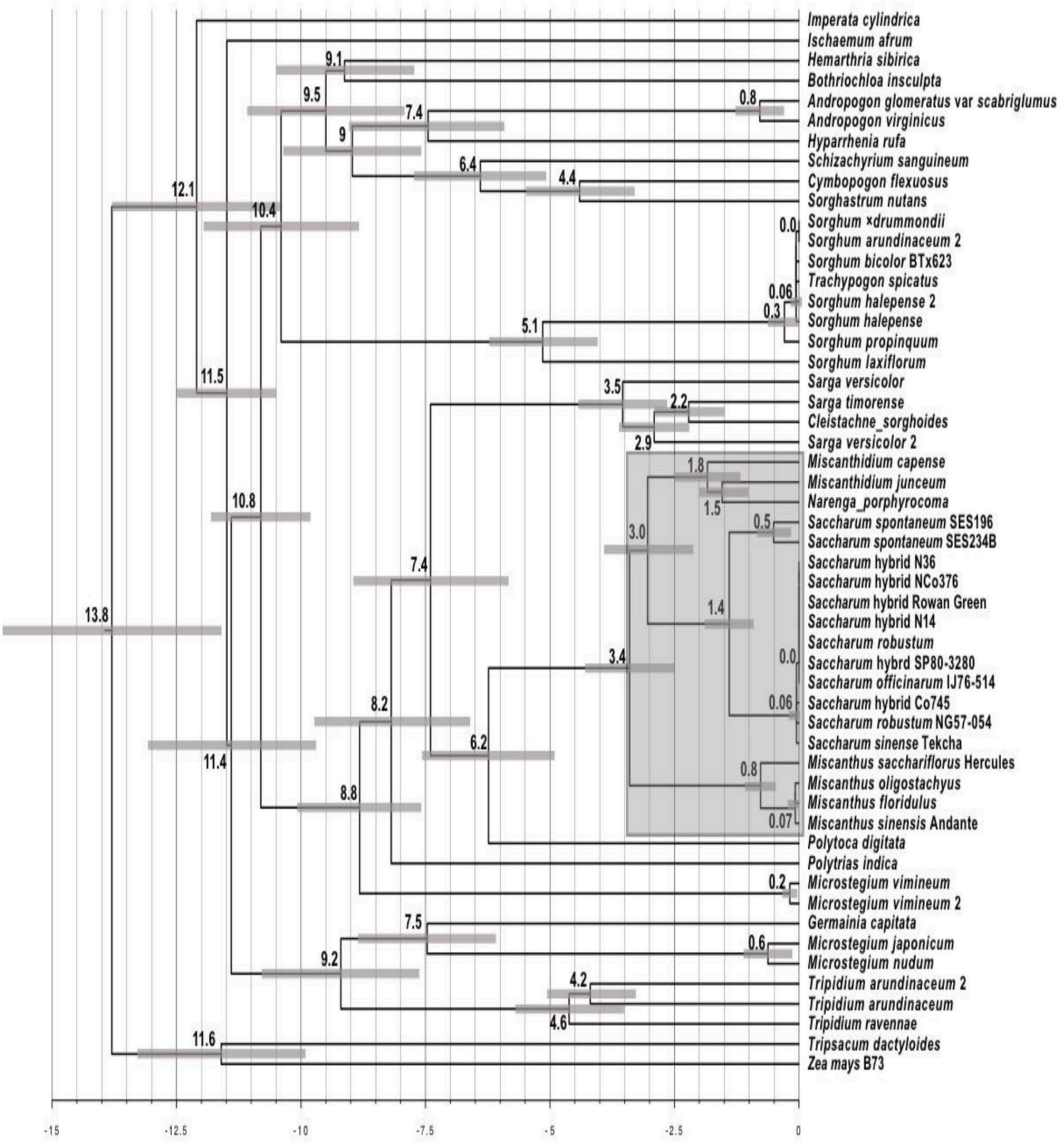
Chronogram derived from the alignment of Andropogoneae ITS cassette sequences. The chronogram was generated with r8s from the Maximum Likelihood ITS phylogeny from Figure 3. The scale at the bottom represents millions of years before present. Numbers at nodes represent the age of that node as millions of years before present. Scale bars at nodes represent the central 95% of the age distribution (i.e., 95% confidence interval) as determined by bootstrap resampling. The shaded region centered on *Saccharum* represents the 3.4 million year window in which wild hybridizations between *Saccharum* and other genera is possible.

The Maximum Likelihood phylogeny was converted into a chronogram (Figure 4) using r8s (Sanderson, 2003) with 95% branch confidence values determined by re-analyzing the non-parametric bootstrap tree set generated by IQ-Tree. Broadly, timings are consistent with previous work (Estep et al., 2014; Lloyd Evans and Joshi, 2016a) with only the genera *Miscanthus* and *Miscanthidium* lying within the 3.4 million year window where wild hybridization is possible as determined by Lloyd Evans and Joshi (2016a) when analyzing wild (i.e., not human mediated) hybridization within the Andropogoneae, specifically the Saccharinae. As it is placed within *Sarga, C. sorghoides* is the only other South African genus (apart from *Miscanthidium*) that lies within the 7.4 million year window chosen as a divergence cut-off for this project.

### Spatial assessment within the sugarcane cultivation region

*Imperata cylindrica, S. arundinaceum*, and *M. capense* showed the highest prevalence within sugarcane cultivation areas (Table 6). Three species from Sorghinae, namely *C. sorghoides, S. nudipes*, and *Sorghum* × *drummondii* showed low prevalence within sugarcane QDS (Table 6). The highest spatial overlap of wild relatives with QDS containing sugarcane plantations revealed a similar outcome to the prevalence rankings (Table 7). In both cases, i.e., prevalence and spatial overlap, the highest and lowest score values differed substantially. *I. cylindrica* showed the highest likelihood for spatial congruence with sugarcane and *S. nudipes* the least.

**Table 6:** Prevalence or commonness of individuals (based on herbarium specimens) of *Saccharum* wild relatives in sugarcane cultivation areas.

**Species**	**No. individuals within sugarcane QDS**	**Proportion of individuals within sugarcane QDS**	**No. individuals bordering sugarcane QDS**	**Proportion of individuals bordering sugarcane QDS**	**Total proportion of sugarcane QDS + bordering sugarcane QDS**	**Score**
*Cleistachne sorghoides*	2	1	3	2	3	3
*Imperata cylindrica*	99	33	31	25	58	11
*Microstegium nudum*	10	3	14	11	14	5
*Miscanthidium capense*	35	12	14	11	23	9
*Miscanthidium junceum*	15	5	13	11	16	7
*Sarga versicolor*	10	3	20	16	19	8
*Sorghastrum nudipes*	1	0.3	1	1	1.3	1
*Sorghastrum stipoides*	26	9	8	7	16	7
*Sorghum arundinaceum*	85	28	14	11	39	10
*Sorghum* × *drummondii*	3	1	1	1	2	2
*Sorghum halepense*	16	5	4	3	8	4

*Calculation of scores was based on ranking the commonness of species from highest to lowest, with most common species scoring 11 and least common receiving 1*.

**Table 7:** Spatial overlap (shared occurrence) of *Saccharum* wild relatives (based on herbarium specimens) with sugarcane cultivation areas (113 QDS).

**Species**	**Sugarcane QDS**	**Overlapping %**	**Score**
*Cleistachne sorghoides*	2	2	2
*Imperata cylindrica*	38	34	11
*Microstegium nudum*	5	4	4
*Miscanthidium capense*	19	17	9
*Miscanthidium junceum*	10	9	5
*Sarga versicolor*	15	13	7
*Sorghastrum nudipes*	1	1	1
*Sorghastrum stipoides*	14	12	6
*Sorghum arundinaceum*	35	31	10
*Sorghum* × *drummondii*	3	3	3
*Sorghum halepense*	16	14	8

*Calculation of scores was based on ranking species occurrences from highest to lowest, with highest ranked species being scored 11 and lowest scoring 1*.

No collections or observations were made of five wild relatives within sugarcane fields within 700 m of the field margin (Table 8). These species can therefore not be considered as common weeds of sugarcane plantations besides the prevalence and spatial overlap with some sugarcane QDS. In general, members of *Sorghum* scored higher rankings for proximity to sugarcane plantations, except for *Sarga versicolor* (Table 8), and this is ascribed to preferences for habitat associated with sugarcane fields. *Imperata cylindrica* also ranked high, indicating its ability to colozise sugarcane fields. *Miscanthidium* species were moderately associated with sugarcane fields (Table 8). Both *I. cylindrica* and *M. capense* were found to be weeds in sugarcane plantations during field surveys although these species were not documented in South African literature as such.

**Table 8:** Proximity or closeness of *Saccharum* wild relatives (based on herbarium specimens, field observations and literature) to sugarcane fields in cultivation areas.

**Species**	**Recorded from field and margins (fm)**	**Literature confirmations (li)**	**fm + li**	**Score**
*Cleistachne sorghoides*	–	–	Absent	0
*Imperata cylindrica*	7	1	8	10
*Microstegium nudum*	–	–	Absent	0
*Miscanthidium capense*	3	–	3	7
*Miscanthidium junceum*	1	–	1	6
*Sarga versicolor*	–	–	Absent	0
*Sorghastrum nudipes*	–	–	Absent	0
*Sorghastrum stipoides*	–	–	Absent	0
*Sorghum arundinaceum*	25	2	27	11
*Sorghum* × *drummondii*	3	1	4	9
*Sorghum halepense*	3	1	4	9

*Calculation of scores was based on ranking species proximity to fields from highest to lowest, with highest ranked species being scored 11. A score of 0 was given when no records could be found and therefore proximity data is not currently known (absence equates to no ranking)*.

*Imperata cylindrica, M. junceum*, and *S. arundinaceum* were ranked highest in terms of having extensive road and railway networks associated with their QDS of occurrence (Table 9). These networks present a higher likelihood for these species to spread into and within sugar cultivation areas compared with species that have fewer distribution networks. Species that are in isolated QDS and that are normally restricted to certain locations will also lack these distribution networks.

**Table 9:** Distribution potential of *Saccharum* wild relatives (based on road and railway networks) in sugarcane cultivation areas.

**Species**	**QDS with railway line (rl_1_)**	**QDS with railway line bordering (rl_2_)**	**QDS with roads (rd_1_)**	**QDS with roads bordering (rd_2_)**	**rl_1_ + rl_2_ + rd_1_ + rd_2_**	**Rank**
*Cleistachne sorghoides*	6	14	7	28	55	1
*Imperata cylindrica*	49	65	85	165	364	11
*Microstegium nudum*	7	29	11	55	102	5
*Miscanthidium capense*	25	38	41	77	181	7
*Miscanthidium junceum*	36	63	59	161	319	10
*Sarga versicolor*	18	29	37	101	185	8
*Sorghastrum nudipes*	5	8	8	44	65	2
*Sorghastrum stipoides*	12	26	18	37	93	4
*Sorghum arundinaceum*	28	57	60	164	309	9
*Sorghum* × *drummondii*	4	20	6	42	72	3
*Sorghum halepense*	16	36	21	85	158	6

*Calculation of scores was based on ranking species from highest to lowest using the number of roads and railways present in the grids of wild relatives, and scoring the largest network as 11 and the smallest 1*.

### Gene flow likelihood

**Figure 5:**
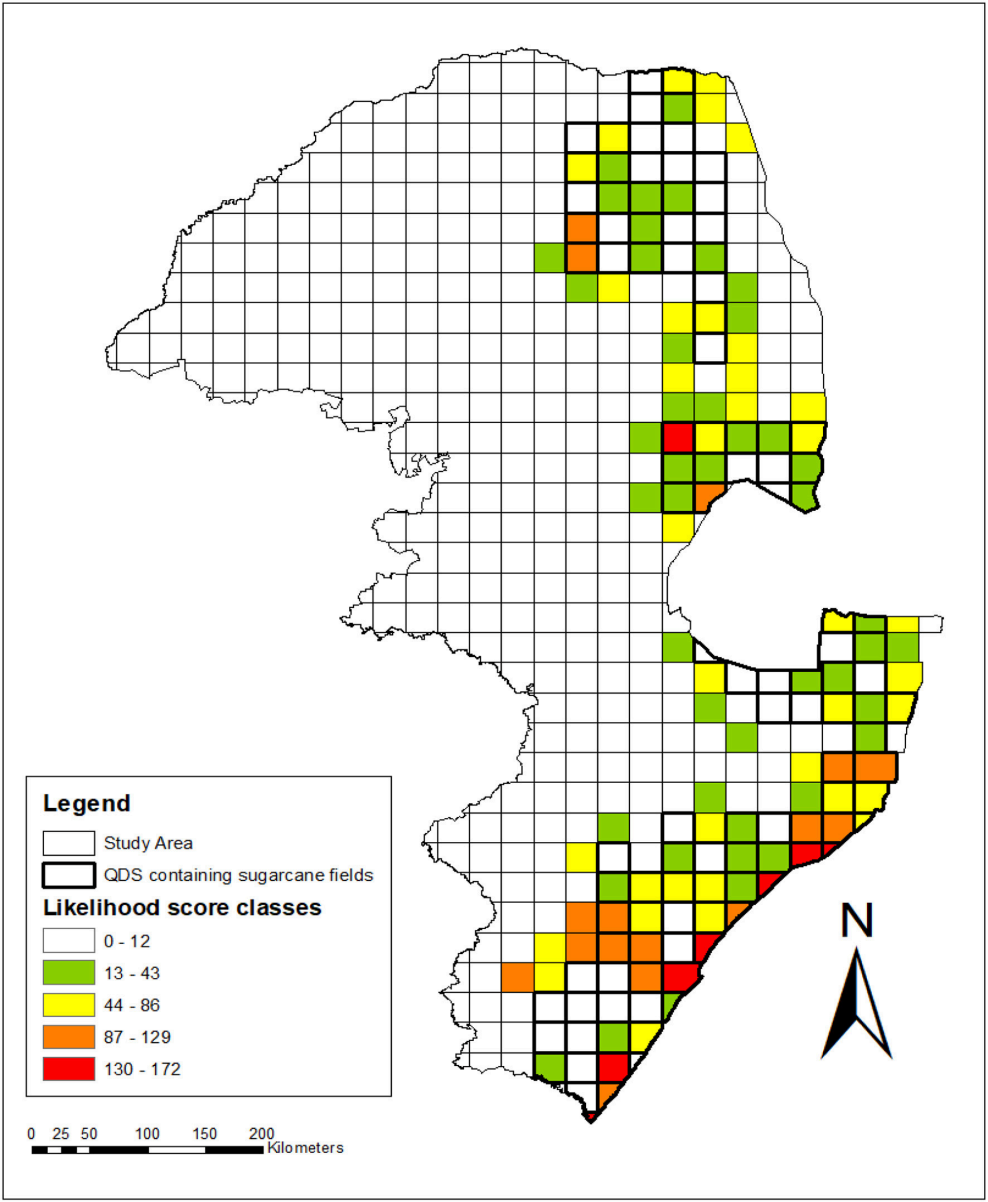
Spatial, temporal and relatedness assessment indicating the levels of likelihood for gene flow to occur between sugarcane and wild relatives in the sugar production region of South Africa. Grid values were calculated by summing the likelihood scores allocated per species (from Table 10) for all the species recorded per grid. QDS with sugarcane plantations are indicated with bold lines, whereas other QDS of the study area without sugarcane plantations are not shown with bold lines. Likelihood for gene flow: *Sorghastrum nudipes* scored 6 and there was no sugarcane QDS containing only this wild relative species. QDS with sugarcane plantations without wild relatives (0–12); sugarcane QDS plantations with wild relatives: very low (13–43); low (44-86); high (87–129); very high (130–172).

*Imperata cylindrica* scored the highest during the spatial and temporal assessment, followed by *S. arundinaceum* and *M. capense* (Table 10). *M. junceum, Sorghum* × *drummondii*, and *S. halepense* are further species with high scores. However, based on the relatedness assessment, *I. cylindrica* and the above *Sorghum* species are not closely related with commercial sugarcane (Figure 2) and are therefore not candidates to consider for gene flow. A likelihood score based on spatial, temporal and relatedness assessments (Figure 5) highlighted the two *Miscanthidium* species. Although *S. arundinaceum* had the highest overall score its distance from *Saccharum* in the phylogeny generated in our study makes it low risk for out crossing. Species with low scores are not considered to present any likelihood for gene flow, especially if these species have diverged from *Saccharum* at more than 7.3 million years (e.g., *Sorghum*).

**Table 10:** Score per species calculated by equal weighting of factors obtained for each of spatial (prevalence, spatial overlap, proximity, and distribution potential), temporal (flowering time), and relatedness [hybridization and phylogenetics (Figure 3)] assessments.

**Species**	**Prevalence**	**Spatial overlap**	**Proximity**	**Distribution potential**	**Spatial assessment**	**Temporal assessment**	**Hybridization**	**Phylogenetics**	**Relatedness assessment**	**Likelihood score S:T:R (1:1:2)**
*Sorghum arundinaceum*	10	10	11	9	10	11	11	6	8.5	38
*Miscanthidium capense*	9	9	7	7	8	9	8	11	9.5	36
*Miscanthidium junceum*	7	5	6	10	7	7	8	11	9.5	33
*Sorghum* × *drummondii*	2	3	9	3	4	9	11	6	8.5	30
*Imperata cylindrica*	11	11	10	11	11	11	7	1	4	30
*Sorghum halepense*	4	8	9	6	7	7	9	6	7.5	29
*Microstegium nudum*	5	4	0	5	4	7	0	7	3.5	18
*Sarga versicolor*	8	7	0	8	6	3	0	9	4.5	18
*Sorghastrum stipoides*	7	6	0	4	4	7	0	3	1.5	14
*Cleistachne sorghoides*	3	2	0	1	2	2	0	9	4.5	13
*Sorghastrum nudipes*	1	1	0	2	1	2	0	3	1.5	6

*Gene flow likelihood score was calculated by weighting the spatial, temporal, and relatedness assessments at 1:1:2*.

Closely related species with high spatial congruity pose the highest likelihood for gene flow and certain areas can be flagged where this is the case. No sugarcane QDS with very high likelihood for gene flow was found in Limpopo but there were two of high likelihood in Modjadjiskloof and Tzaneen (Figure 5). There was one QDS with very high likelihood in Nelspruit in addition to one QDS with high likelihood in Mpumalanga province. Thirteen QDS with high and 7 with very high likelihood were identified for KwaZulu-Natal, namely Durban, Felixton, Gingindlovu, Port Edward, Port Shepstone, Richards Bay, and Verulam. Overall it appears as if coastal and southern-inland KwaZulu-Natal have the highest likelihood for gene flow to occur based on relatedness, temporal and spatial congruity (Figure 5).

## Discussion

Several studies have assessed the potential hybridization between plants and their closest relatives in GM scenarios (Ellstrand et al., 1999; FitzJohn et al., 2007; McGeoch et al., 2009) and similar evaluations have been made in sugarcane (Bonnett et al., 2008; Cheavegatti-Gianotto et al., 2011; Organisation for Economic Cooperation and Development, 2013). Our study was designed to consider these factors in a South African context. A review by Ellstrand et al. (1999) listed sugarcane amongst the world's important crop species which hybridize with wild relatives in agricultural systems. Commercial sugarcane cultivars have not been reported to spontaneously hybridize with any related genera and in the two published reviews that assessed the likelihood of GM sugarcane outcrossing with wild species there was no evidence of natural hybridization (Bonnett et al., 2008; Cheavegatti-Gianotto et al., 2011).

*Imperata, Sorghum, Narenga*, and *Zea* are genera found in South Africa that have been artificially crossed with sugarcane, and evidence of introgression has been confirmed on a molecular level (except in *Imperata*) (Bonnett et al., 2008; Hodnett et al., 2010). It was evident that sugarcane has a considerably low success of producing hybrids compared with its progenitors (i.e., *Saccharum officinarum*) (Piperidis et al., 2000; Aitken et al., 2007). Cheavegatti-Gianotto et al. (2011) noted that even when the barriers to hybridization were eliminated in artificial crosses (i.e., where flowering was synchronized, male pollen viability was increased and numerous florets were hand pollinated), there was poor growth and low survival in seedlings of the progeny. Even though *Saccharum* has previously crossed with *Sorghum* and *Miscanthidium* (Bourne, 1935; Brett, 1954; Gupta et al., 1978), Bonnett et al. (2008) concluded that these genera are unlikely to interbreed either spontaneously or without intervention from breeders due to the low survival rate of the seedlings.

Although the spatial assessment, both prevalence and spatial overlap, confirmed that *I. cylindrica, S. arundinaceum*, and *M. capense* had the highest spatial congruence within sugarcane cultivation areas (Tables 4, 6–8), and synchronous flowering times could facilitate gene flow (Table 5), evidence gathered in the present study using phylogenetic analyses of the ITS cassette demonstrated that commercial sugarcane cultivars were sister to *Miscanthidium* species and *Narenga*, but were only distantly related to *S. arundinaceum* and *I. cylindrica* (Figure 3).

It is generally accepted (Kellogg, 2013) that the “core” Andropogoneae (Figure 3) defines the dividing line between species that could be part of the Saccharinae and those that are not. Our phylogeny (Figure 3) clearly places *I. cylindrica* and *Ischaemum afrum* outside the Saccharinae. The same is true for genus *Tripidium* (Asiatic species). We also place *Sorghum* as sister to the core Andropogoneae (as has also been reported by Hawkins et al., 2015). This means that *Sorghum* is over 11 million years distant from *Saccharum*; well outside the natural hybridization window. *Polytrias indica* and *M. vimineum* form outgroups to the core Saccharinae. *Sarga* is sister to the core Saccharinae, but this is essentially an Asiatic genus; the one exception being *C. sorghoides*, which is native to Eastern Africa from Mpumalanga to Ethiopia (Clayton et al., 2006). However, with a base chromosomal number of 9 (Celarier, 1958), *Cleistachne* is unlikely to be karyotypically compatible with sugarcane.

*Miscanthus* and *Polytoca*, which are sister to *Saccharum* are Asiatic species as well. The next grouping, which is directly sister to *Saccharum sensu stricto* includes the African *Miscanthidium* species as well as *Narenga porphyrocoma*, which is mainly Asiatic, but has a rump population in Ethiopia. In an African context, at least in terms of evolutionary distance, these are the species most likely to hybridize with *Saccharum. Narenga–Saccharum* hybrids have been generated in breeding programmes, but they tend to be male sterile and suffer chromosomal loss in the F2 generation (Price, 1957). Chloroplast data (D Lloyd Evans, personal communication) indicates that *Narenga* hybridized with *Saccharum* more recently than *Miscanthidium*, and thus may contain more compatible chromosomes.

*Miscanthidium* species have a base chromosome number of 15 and show no recent hybridization with sugarcane (the two genera have been isolated for at least 2.5 million years). Thus it is likely that *Miscanthidium* and *Saccharum* are not chromosomally compatible. As an Asiatic and Ethiopian species, *S. narenga* poses no threat to gene flow with South African sugarcane, but could be a bridge species in a broader African context. It should be noted however, that of all the genera presented in the phylogeny (Figure 3) only the Asiatic and Polynesian species, *Miscanthus floridulus* has categorically been demonstrated to have hybridized with *Saccharum* in the wild (Lloyd Evans and Joshi, 2016a).

As sugarcane hybrids are based on a small number of inter-related parental lines, it is hardly surprising that these cultivars could not be resolved in the ITS phylogeny and the ITS cassette itself does not possess sufficient characters to resolve recently diverged species or cultivars. However, we see that the two *S. spontaneum* accessions are clearly divergent from the other *Saccharum* species or cultivars. *S. sinense* cv Tekcha emerges as ancestral to the remaining *Saccharum* species with 100% support. This is not unexpected as *S. sinense* accessions are ancient hybrids of *S. officinarum* and *S. spontaneum* (Irvine, 1999). As a grouping, *S. robustum* NG57-054, *Saccharum* hybrid cv Co745 and *S. officinarum* IJ76-514 were also resolved from the sugarcane hybrids with 100% support, though resolution within the monophyletic grouping was not possible.

The chronogram (Figure 4) provides timings for the radiation events undergone by species analyzed in this study. Few genera lie within the 3.4 million year window where wild hybridization is possible between *Saccharum* and other genera. Even if this window is extended to 7.4 million years, this only adds an additional two genera. All members of *Sorghum* (including *Trachypogon spicatus*) can be excluded as they are 10.4 million years divergent from *Saccharum*. The same applies to *I. cylindrica*, which is 12.1 million years divergent. Interestingly, the chronogram places *Tripidium* species (which sugarcane breeders have been attempting to introgress into *Saccharum* hybrid cultivars for over 50 years with poor success) as 11.4 million years divergent from *Saccharum*. The Southern African species, *C. sorghoides* lies within the genus *Sarga* which is 7.4 million years divergent from sugarcane. However, this species poses low risk of hybridization as it lies outside the wild hybridization window. The only species of high concern in terms of divergence times from *Saccharum* are those within the genus *Miscanthidium*, most especially *M. capense*, and *M. junceum* which are estimated to be approximately 3 million years divergent from *Saccharum* (Figure 4).

An unexpected finding was that commercial sugarcane cultivars N36 and N14 had pollen viability of up to 80% in some regions of South Africa (Figure 2). Even though no similar studies conducted field assessments across the sugarcane cultivation regions in South Africa, sugarcane seldom produces viable pollen under natural conditions at Mount Edgecombe (site 8) (Brett, 1950; Horsley and Zhou, 2013). Pollen viability gradually decreased from the northern inland (85%) to the south coastal regions (0%) of the study. Within certain study sites (e.g., site 5), some cultivars showed pollen viability of 70%, while others had <10%. A similar study in Brazil reported 100% viable pollen in some cultivars while others showed pollen viability of <9%, under the same environmental conditions (Melloni et al., 2015). Pollen viability has also been closely associated with genotype (Nair, 1975; Pagliarini, 2000; Melloni et al., 2015).

There is a higher likelihood for gene flow when potential pollen recipients flower at the same time as donor crop species when they are in close proximity (Ellstrand et al., 1999; Chapman and Burke, 2006; Schmidt and Bothma, 2006; FitzJohn et al., 2007; Bonnett et al., 2008; Tesso et al., 2008; Nieh et al., 2014). In the current study, there is only one related species with flower synchrony and shared habitat, *M. capense*, which presents the highest potential for gene flow (Table 10 and Figure 5). Although, as discussed previously, all verified hybrids between sugarcane and numerous species within the Andropogoneae have been created through human mediation. Moreover, in all cases hybrids are typically male sterile (Bremer, 1961; Kandasami, 1961; Aitken et al., 2007; Sobhakumari and Nair, 2014) and in F2 and subsequent generations there is considerable chromosomal loss. Thus no sugarcane hybrid reported thus far is a true hybrid, they are always intergeneric (partial) hybrids. Primarily this is due to chromosome number incompatibility (Figures 3, 4) and reflects the divergent evolutionary history of the major lineages within the Andropogoneae. Whilst there are reports of possible hybridizations between *Saccharum* species and related species in the wild, there have been no reports of wild hybridizations with modern hybrid sugarcane cultivars (Cheavegatti-Gianotto et al., 2011). Again this is an issue of chromosomal compatibility. Wild type *Saccharum officinarum* has a base chromosome count of 60 or 80 (typically the latter), but modern hybrids have a chromosome count of about 136 chromosomes—this is variable in different hybrids, but there are typically 10% *S. spontaneum* chromosomes and 90% *S. officinarum* chromosomes (Bremer, 1961). As a consequence, chromosomal incompatibility is far more likely between modern commercial sugarcane hybrids and wild species than between sugarcane's ancestors and wild species. Indeed, even back crosses of commercial hybrids with their immediate ancestors (*S. spontaneum* and *S. officinarum*) often lead to problems of male sterility (Babu, 1990). For crosses between sugarcane hybrid and wild species of low ploidy, not only is there an issue of chromosome incompatibility due to evolutionary distance, there is the added problem of lack of meiotic pairing due to differential chromosome numbers.

In our study, *I. cylindrica, M. capense, M. junceum, S. arundinaceum, S*. × *drummondii*, and *S. halepense* were found in relatively close proximity to sugarcane fields (Supplementary Figure 1). The latest review of invasive grasses of South Africa (Visser et al., 2017) reported *Sorghum* × *drummondii* and *S. halepense* amongst 256 weedy grasses that were introduced to agricultural systems. Weedy relatives may be considered as higher risk for gene flow potential when they are geographically associated with GM crops (Bonnett et al., 2008; Organisation for Economic Cooperation and Development, 2013). In general, most problematic weeds of sugarcane are in the Andropogoneae (Cheavegatti-Gianotto et al., 2011; Organisation for Economic Cooperation and Development, 2013). *Imperata cylindrica* and members of *Sorghum* have been documented as aggressive weeds of agricultural fields including sugarcane plantations in many countries (Van Oudtshoorn, 1999; Firehun and Tamado, 2006; Bonnett et al., 2008; Organisation for Economic Cooperation and Development, 2013; Takim et al., 2014). *Sorghum arundinaceum* and *S*. × *drummondii* are considered as weeds of sugarcane in South Africa (Van Oudtshoorn, 1999; Milton, 2004; Fish et al., 2015). Studies from Nigeria reported *I. cylindrica* amongst problem weeds of sugarcane (Takim et al., 2014), and both *S. arundinaceum* and *S*. × *drummondii* are regarded as major weeds of sugarcane in Ethiopia (Firehun and Tamado, 2006). For South African situations assessed in this study, although *M. capense* and *M. junceus* may be considered to be weeds in sugarcane fields, they are not considered to be “weedy”^7^.

Vehicles are amongst the main factors associated with the spread of weedy grasses in South Africa (Milton, 2004). The transport network therefore gives an indication of the potential for weedy relatives of sugarcane to spread, with denser networks implying higher chances for migrations. Furthermore, sugarcane relatives are often associated with roadsides as a preferred habitat (Retief and Herman, 1997; Van Oudtshoorn, 1999; Fish et al., 2015). Potential distribution networks of related species in our study show that most would be able to spread from the areas in which they are found, for example, *M. capense* is associated with vast road and rail networks (Table 9), which suggests that anthropogenic activities can enhance seed dispersal and increase gene flow potential (Andow and Zwahlen, 2006) in weedy species.

## Conclusions

Phylogenetic analyses of the ITS cassette showed that the closest wild relative species to commercial sugarcane were *M. capense, M. junceum*, and *N. porphyrocoma. Sorghum* was found to be more distantly related to *Saccharum* than previously described. Similarly, *Imperata* is so distant from *Saccharum* that it poses no risk of gene flow. In the wild, no hybrids between modern sugarcane hybrid cultivars and any species have been reported. All documented wild hybrids are between sugarcane's ancestors (*S. officinarum, S. robustum*, and *S. spontaneum*) and a small number of closely related species. The phytogeography assessment indicated that the only wild relatives likely to be recipients of gene flow in the study area are *Miscanthidium* species—*M. capense* was observed to be a weed in cultivated sugarcane plantations but it does not have characteristics that make it “weedy.” Consequently, even although some commercial sugarcane cultivars do produce fertile pollen—especially in northern irrigated areas of KZN, there is a low likelihood of hybrids occurring in the natural environment. Therefore in a future scenario where GM sugarcane is cultivated in South Africa, the risk of gene flow to wild relatives is low.

## Author contributions

SaS conceived the idea and acquired funding for this research. SB and StS designed the experiments. HK and DK conducted the experimental work, analyzed data and interpreted results for their MSc degrees at North-West University, South Africa. Supervision was provided by SB and StS and co-supervision by JvdB, DC, and SaS. SaS co-ordinated the paper writing and submission. DLE performed assemblies from short read data, generated the alignments, performed and interpreted the phylogenies. All authors reviewed, revised, and approved the final version of the manuscript.

### Conflict of interest statement

Author DLE acts as an unpaid Senior Informatics Specialist for BeauSci Ltd. The relationship is based on data sharing only. The remaining authors declare that the research was conducted in the absence of any commercial or financial relationships that could be construed as a potential conflict of interest.

## Footnotes

^1^http://www.isaaa.org/

^2^https://www.ispotnature.org

^3^https://ncbi.nlm.nih.gov/sra

^4^http://purl.org/phylo/treebase/phylows/study/TB2:S22812

^5^http://www.sasa.org.za/sasri

^6^http://www.timeanddate.com

^7^http://www.hear.org/gcw
