# Supplementary figures and images for: Assessing the Likelihood of Gene Flow From Sugarcane (Saccharum Hybrids) to Wild Relatives in South Africa
Source: Front Bioeng Biotechnol. 2018 Jun 7;6:72. doi: 10.3389/fbioe.2018.00072 (PMC5999724; doi:10.3389/fbioe.2018.00072)

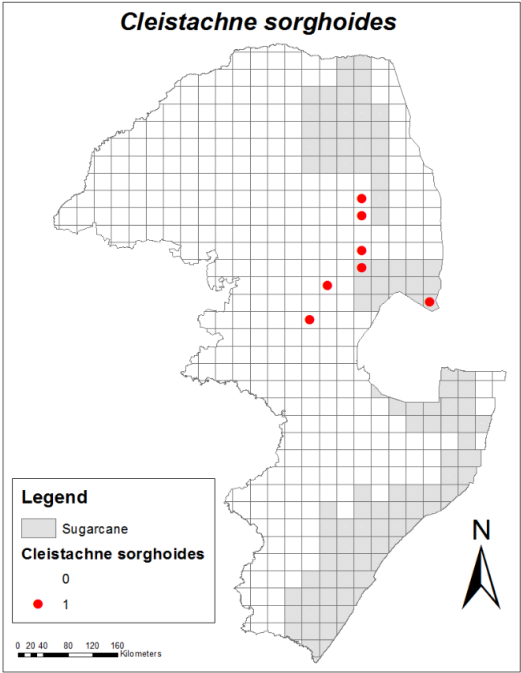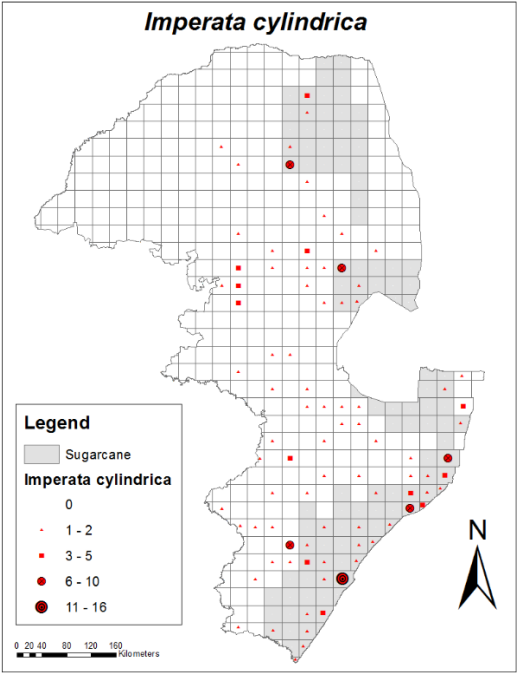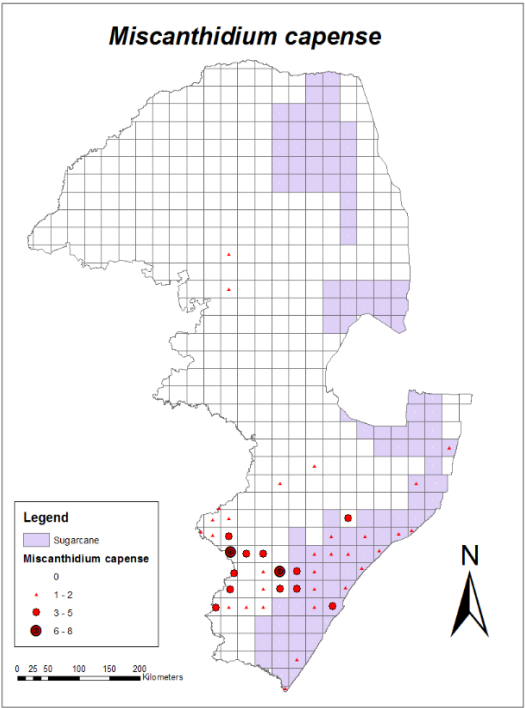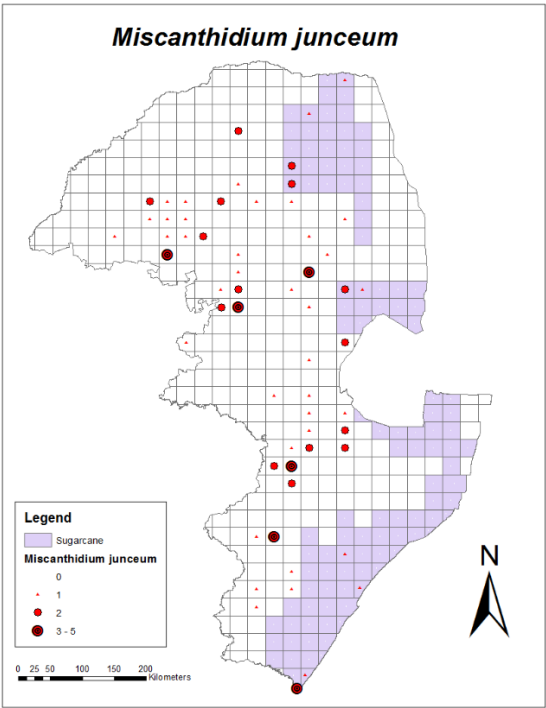

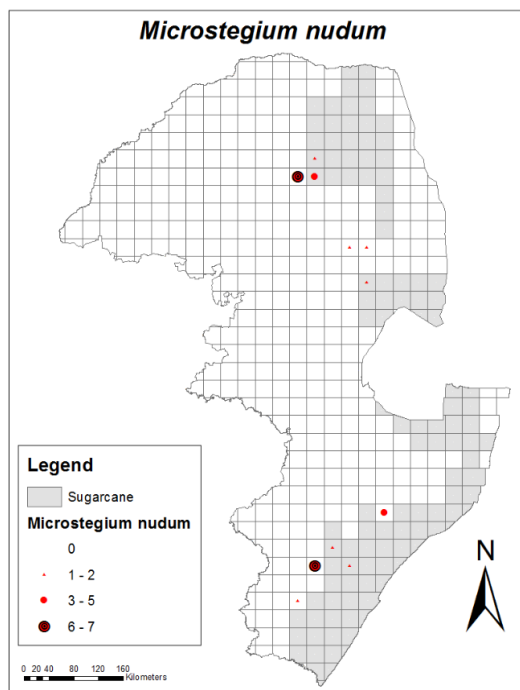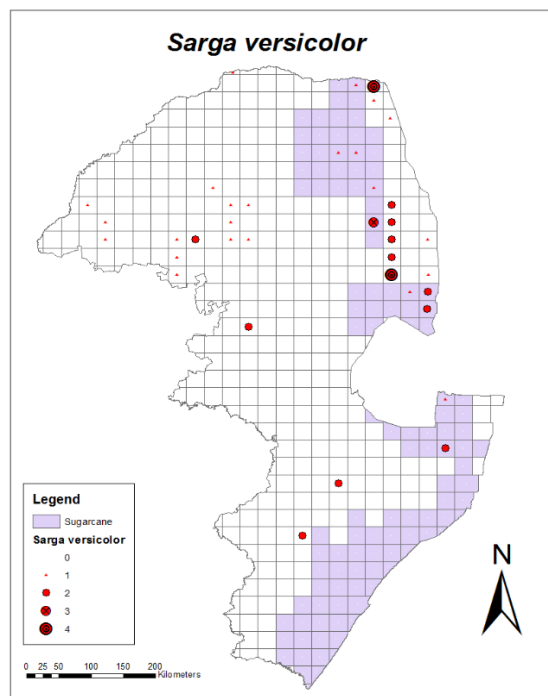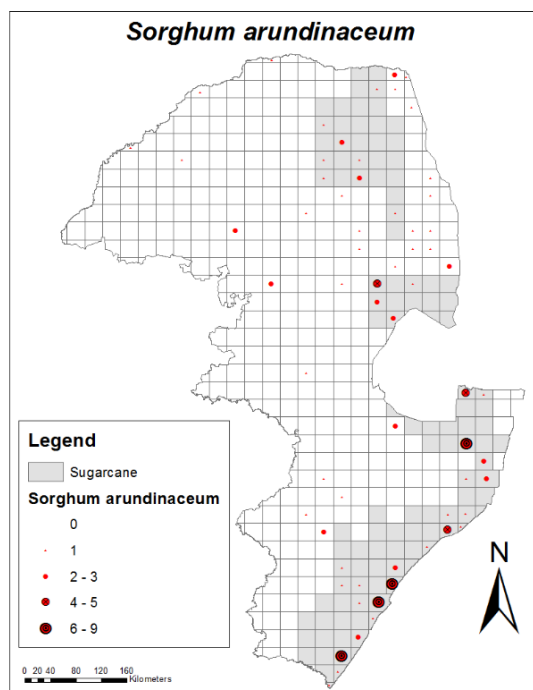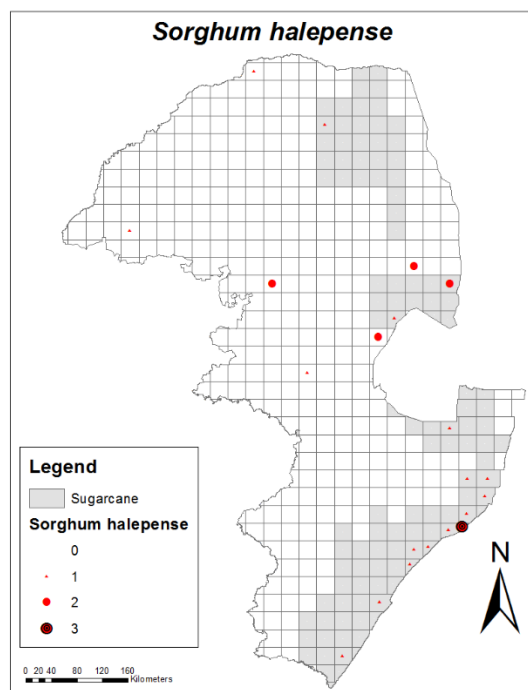

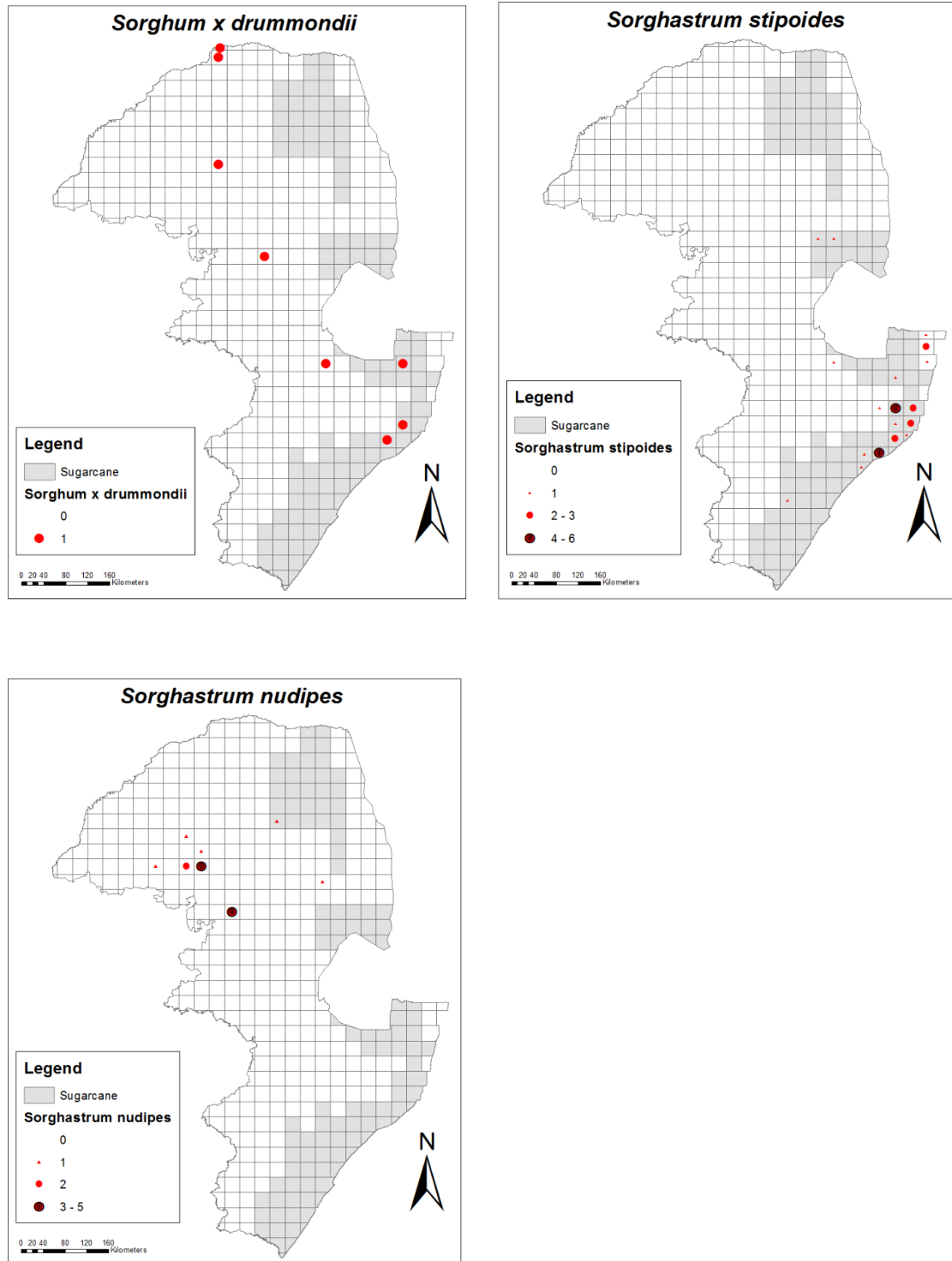

**Supplementary Figure 1.** Distribution map of each *Saccharum* hybrid wild relative.

Supplement: Supplementary file 2 [file Image_1.pdf]
